# Unraveling the pleiotropic effects of CCR2-dependent signal transduction in fibrosis development

**DOI:** 10.7150/thno.130426

**Published:** 2026-04-08

**Authors:** Guozheng Sun, Jiaxing Wang, Jiayou Tang, Mingzhe Chen, Zhe Zhang, Huadong Zhao, Zhenxiao Jin, Xuezeng Xu, Yang Yang, Jincheng Liu

**Affiliations:** 1 Department of Cardiovascular Surgery, Xijing Hospital, The Airforce Medical University, 127 Changle West Road, Xi'an 710032, China.; 2 Xi'an Key Laboratory of Innovative Drug Research for Heart Failure, Faculty of Life Sciences and Medicine, Northwest University, 229 Taibai North Road, Xi'an 710069, China.; 3 Department of General Surgery, Tangdu Hospital, The Airforce Medical University, 1 Xinsi Road, Xi'an 710038, China.

**Keywords:** CCR2, fibrosis, regulatory network, diagnostic biomarkers, targeted therapy

## Abstract

Fibrosis is a pathological process characterized by the abnormal deposition of connective tissue across multiple organ systems. Given the high prevalence of fibrotic diseases and the limited availability of clinical treatment options, it has emerged as a major challenge in contemporary medicine. Chronic inflammation is widely recognized as a common pathological basis of various fibrotic disorders. In fibrosis progression, CCR2 acts as a critical signaling hub, initiating cascade reactions and contributing to the formation of a complex regulatory network. Studies have demonstrated that in most organ fibrotic processes, CCR2 primarily exerts pro-fibrotic effect by recruiting inflammatory monocytes, activating fibroblasts, and promoting extracellular matrix deposition. However, the function of CCR2 is not unidimensional. It may also play a regulatory role in promoting fibrosis regression under specific tissue and pathological contexts. CCR2 signaling exhibits dual regulatory properties at different stages of liver fibrosis. CCR2 promotes injury in the early phase, while participating in fibrosis reversal by mediating macrophage transition toward a reparative phenotype and facilitating extracellular matrix degradation. This stage-dependent behavior suggests that inappropriate timing of intervention may disrupt repair process, and the functional redundancy of the chemokine system may trigger compensatory adaptations. Together, these factors constitute the core translational challenges facing CCR2-targeted therapeutic strategies. This article systematically reviews the complex regulatory network and pivotal role of CCR2 signaling in fibrosis progression, summarizes the latest advances in the diagnosis and treatment of clinically relevant fibrotic diseases associated with this pathway, analyzes the specific challenges in translating CCR2-targeted therapies into clinical practice, and outlines future research directions.

## 1. Introduction

Fibrosis is generally considered to be the result of dysregulated tissue repair responses caused by chronic inflammation, with excessive accumulation of extracellular matrix (ECM) proteins as its primary characteristic 1. Hypoxia, viral infections, allergies, and other factors can all lead to tissue damage 2, 3. In response to tissue damage, fibroblasts from various sources initiate the healing response by remodeling the extracellular environment to restore tissue integrity. Typically, this profibrotic process shuts down once the tissue has healed 4. However, persistent inflammatory injury can cause this repair response to become excessive or uncontrolled, leading to excessive ECM deposition, which in turn results in organ structural damage and functional decline 5. Fibrosis occurs in almost all organs and tissues of the human body, including the liver, lungs, heart, and kidneys 6-9. Among these, idiopathic pulmonary fibrosis (IPF) is a chronic progressive disease with variable disease course and high mortality rate 10. Currently, clinical treatment options for IPF are limited, with no significantly effective therapeutic drugs available, and treatment can only partially slow the progression of IPF and reduce the risk of related complications. This also reflects the clinical treatment challenges faced in all fibrosis-related organ and tissue damage.

Although fibrosis in different organs or tissues exhibits specific clinical manifestations and hazards, most fibrotic diseases share a common pathogenic process. Persistent injury stimuli induce inflammatory cell infiltration and chemokine release, which in turn activate fibroblasts, ultimately leading to excessive ECM deposition 11, 12. Within this pathological process, monocyte chemotactic protein-1 (CCL2/MCP-1)/C-C motif chemokine receptor 2 (CCR2) axis occupies a central regulatory position, playing a crucial role in the initiation and progression of fibrosis 13-15. As a key regulator of monocyte/macrophage recruitment, the CCL2/CCR2 axis not only mediates the directed migration of inflammatory cells to sites of injury 16, 17. More importantly, it directly participates in converting initial inflammatory signals into fibrotic effects. Myeloid-derived fibroblast precursors require CCL2/CCR2 signaling to migrate into tissues and differentiate into ECM-producing effector cells 13. Furthermore, CCR2 knockout significantly reduces expression levels of fibrosis markers such as α-smooth muscle actin (α-SMA), fibronectin, and collagen I in the hearts of streptozotocin-induced diabetic cardiomyopathy mice, improving streptozotocin-induced cardiac dysfunction and fibrosis 18. Consequently, CCR2-dependent signaling has emerged as a pivotal link between inflammatory initiation and fibrotic disease progression, making targeted modulation of the CCR2 pathway a promising therapeutic strategy for fibrotic disorders.

In this review, we first introduce the biological functions of CCR2 and CCR2 ligands, and focus on the complex regulatory networks and key roles of CCR2-dependent signaling in the fibrotic process. We then summarize the diagnostic and therapeutic potential of CCR2 signaling, thereby providing valuable insights for future research and clinical practice.

## 2. Basic information on CCR2 and its ligands

### 2.1 Brief introduction of CCR2

CCR2 is a functional chemokine receptor found in various organs and tissues, including the heart, liver, spleen, lungs, kidneys, brain, colon, bladder, skin, and bone marrow. Furthermore, CCR2 is widely expressed in various cell populations, such as monocytes, macrophages, endothelial cells (ECs), lymphocytes, dendritic cells (DCs), and T cells 19-22. Studies have shown that interferon-γ (IFN-γ) acts in concert with bacterial lipopolysaccharide (LPS), tumor necrosis factor α (TNF-α), and interleukin-1β (IL-1β) in activating CCR2 expression 23. Based on the carboxyl-terminal (C-terminal) tail, CCR2 is divided into two subtypes, CCR2A and CCR2B 24, 25. CCR2B is primarily localized on the cell surface, while CCR2A is localized in the cytoplasm 24.

CCR2 belongs to the G protein-coupled receptors (GPCRs) family, with a spatial structure comprising a C-terminal domain, seven helical transmembrane domains connected by intracellular and extracellular hydrophilic loops, and an amino-terminal (N-terminal) domain 26. The N-terminal of CCR2 determines the specificity of ligand binding. CCR2 can bind to multiple CC chemokines, exhibiting significant redundancy. This redundancy is crucial for maintaining the activity and stability of chemokines. CCR2 serves as a high-affinity receptor for multiple members of the MCP family. In humans, CCR2 has four ligands: CCL2 (MCP-1), CCL8 (MCP-2), CCL7 (MCP-3), and CCL13 (MCP-4) 27-29. In mice, CCR2 has three ligands: CCL2 (JE/MCP-1), CCL7 (MCP-3), and CCL12 (MCP-5) 30. Moreover, mouse CCL2 (mCCL2) and CCL12 are the closest homologues to human CCL2, so studying the roles of mouse CCL2 and CCL12 in diseases can reflect the functions of human CCL2 31. CCR2 also binds to cytokine-like 1 (Cytl1) and PC3-secreted microprotein (PSMP)/microseminoprotein (MSMP) 32-34. Among these, Cytl1 has structural similarities with CCL2 and possesses chemotactic activity 32. PSMP is a novel chemokine structurally distinct from CC chemokines, which can mediate monocyte recruitment and migration via activating the CCR2B/extracellular-signal-regulated kinase (ERK) pathway 32, 34.

The C-terminal residues of CCR2 bind to G proteins in CCR2 ligands, thereby activating downstream signaling pathways 26. These signaling events recruit and activate proteins involved in cell transport, thus promoting cell migration along the chemokine gradients. Moreover, CCR2-mediated signaling is recognized as a key coordinator of numerous critical cellular activities, including inflammation, hematopoiesis, wound healing, tumor growth and metastasis, and fibrosis 17, 35-37. Extensive research has shown that CCR2, which binds to CC chemokines, regulates the development of various diseases such as atherosclerosis and myocardial infarction (MI) by promoting bone marrow-derived monocytes mobilization into the bloodstream and migration to inflammatory sites 38, 39. Receptor knockout experiments found that CCR2 knockout (CCR2 ^-/-^) mice exhibit defects in macrophage recruitment, dendritic cell activation, and immune defense functions 40.

### 2.2 Brief introduction to CCR2 ligands

This section primarily introduces members of the MCP family that bind to CCR2 with high affinity, including the discovery, cellular expression, structure, and biological functions of these chemokines **(Table 1)**.

#### CCL2

CCL2, also known as MCP-1, was first isolated from human glioma cells and human blood mononuclear leukocytes 27, 41. Among proteins with similar sequences, the coding regions of human CCL2 and mCCL12 exhibit 68% identity 42. CCL2 is a small molecule protein composed of 76 amino acid residues, secreted by various cells and most abundantly expressed in monocytes, macrophages, lymphocytes, and DCs 43-45. Furthermore, CCL2 expression can be either persistent or inducible. Multiple mediators can induce CCL2 expression, including IL-1β, IL-6, TNF-α, transforming growth factor-β (TGF-β), IFN-γ, granulocyte-macrophage colony-stimulating factor (GM-CSF), and LPS 16, 46-50. Research has demonstrated that CCL2 exhibits significant chemotactic activity toward monocytes, microglia, T cells, natural killer (NK) cells, and fibroblasts 17, 36, 51, 52. CCL2 can also regulate disease progression by modulating the migration and infiltration of these immune cells. CCL2 initiates atherosclerosis by recruiting macrophages and monocytes and promoting these cells migration to the damaged vascular wall 53.

#### CCL7

CCL7, also known as MCP-3, was first discovered in the supernatant of human osteosarcoma cells MG-63 28. At the amino acid level, human CCL7 and CCL2 share 73% structural homology 28. CCL7 is expressed in various cell types, including lymphocytes, DCs, NK cells, astrocytes, and stromal cells 54-57. Under pathological conditions, CCL7 may also be expressed in tumor cells 58. Furthermore, CCL7 is an effective chemotactic agent for various leukocytes, mediating the recruitment and migration of monocytes, macrophages, neutrophils, and eosinophils through interaction with multiple chemokine receptors 59-63. Endothelial dysfunction and vascular lesions in diabetic mice can be effectively alleviated by inhibiting CCL7 64.

#### CCL8

CCL8, also known as MCP-2, was initially identified in the supernatant of human osteosarcoma cells and exhibits 69% structural homology with CCL2 28. Various cell types produce CCL8, including monocytes, fibroblasts, endometrial cells, and mast cells. CCL8 plays a key role in inflammatory responses and allergic diseases by attracting multiple immune cells 65, 66.

#### CCL12

CCL12, also known as MCP-5, is present only in mice and not in humans, and was first identified in allergic pneumonia 67, 68. The mCCL12 protein is 68% identical to human CCL2 protein 31. The chemotactic effects of CCL12 are mediated by binding to the specific receptor CCR2 68.

#### CCL13

Human CCL13, also known as MCP-4, was initially isolated from a human cardiac cDNA library using eotaxin as a probe 29. At the amino acid level, human CCL13 is 65% homologous with human CCL2 29. Various tissues show high levels of CCL13 expression, and this expression increases significantly in tumor cell lines 69, 70. Chondrocytes also secrete large amounts of CCL13, which exacerbates rheumatoid arthritis by promoting fibroblast-like synovial cells proliferation 71. Moreover, CCL13 levels are significantly upregulated under the stimulation of pro-inflammatory cytokines 72, 73. However, the Th2-type cytokine IL-4 inhibits CCL13 expression induced by TNF-α and IL-1β in peripheral blood mononuclear cells, but only minimally affects CCL13 expression in epithelial cells 74.

## 3. Regulatory network of CCR2-dependent signal transduction in fibrosis

Given that CCL2 is the most prominent member in terms of activity among CCR2 ligands, this section will first systematically elucidate the fundamental mechanisms of CCL2/CCR2 signal transduction 17. Existing research indicates that under various internal and external stimuli, the expression of CCR2 and its ligands is finely regulated by multiple upstream factors at different levels. These regulatory factors modulate CCL2 expression through direct or indirect pathways, thereby profoundly influencing the balance between inflammatory responses and tissue repair during fibrosis 75, 76. Accordingly, this section will provide a hierarchical and logically coherent systematic discussion, focusing on the upstream regulatory network, downstream effector pathways, and synergistic molecular interactions of CCR2 signaling in the progression of fibrosis.

### 3.1 CCL2/CCR2 signal transduction

The CCL2/CCR2 axis is the most widely studied mechanism for recruiting monocytes. Upon binding to CCL2, CCR2 undergoes a conformational change, which subsequently activates multiple intracellular G protein-mediated signaling pathways, including the phosphoinositide 3-kinase/ protein kinase B (PI3K/AKT) pathway, mitogen-activated protein kinase (MAPK) pathway, protein kinase C (PKC) pathway, and RAS/RAF/mitogen-activated protein kinase kinase (MEK)/ERK pathway 77-81. These signaling pathways not only participate in cell recruitment and migration processes but also promote the production of various transcription factors and cytokines involved in cell proliferation, growth, and differentiation 82-84. Furthermore, these pathways collectively coordinate biological processes such as cell survival, migration, apoptosis, angiogenesis, and inflammation 79-81, 85, 86. And the CCL2/CCR2 axis is closely associated with the development of various diseases, such as atherosclerosis, stroke, pulmonary arterial hypertension, and cancer 53, 80, 87, 88. Targeting the CCL2/CCR2 axis is considered a key strategy for treating these diseases.

### 3.2 Upstream regulatory factors of the CCR2 signal in the fibrosis process

The CCL2/CCR2 axis is a key signal driving tissue remodeling. We focus on exploring the upstream molecular regulatory network of CCL2. Studies have shown that CCL2 expression and activity are precisely regulated through multi-level, multi-pathway mechanisms, including transcriptional regulation and epigenetic modifications.

#### 3.2.1 Transcriptional regulation

**Figure 1:**
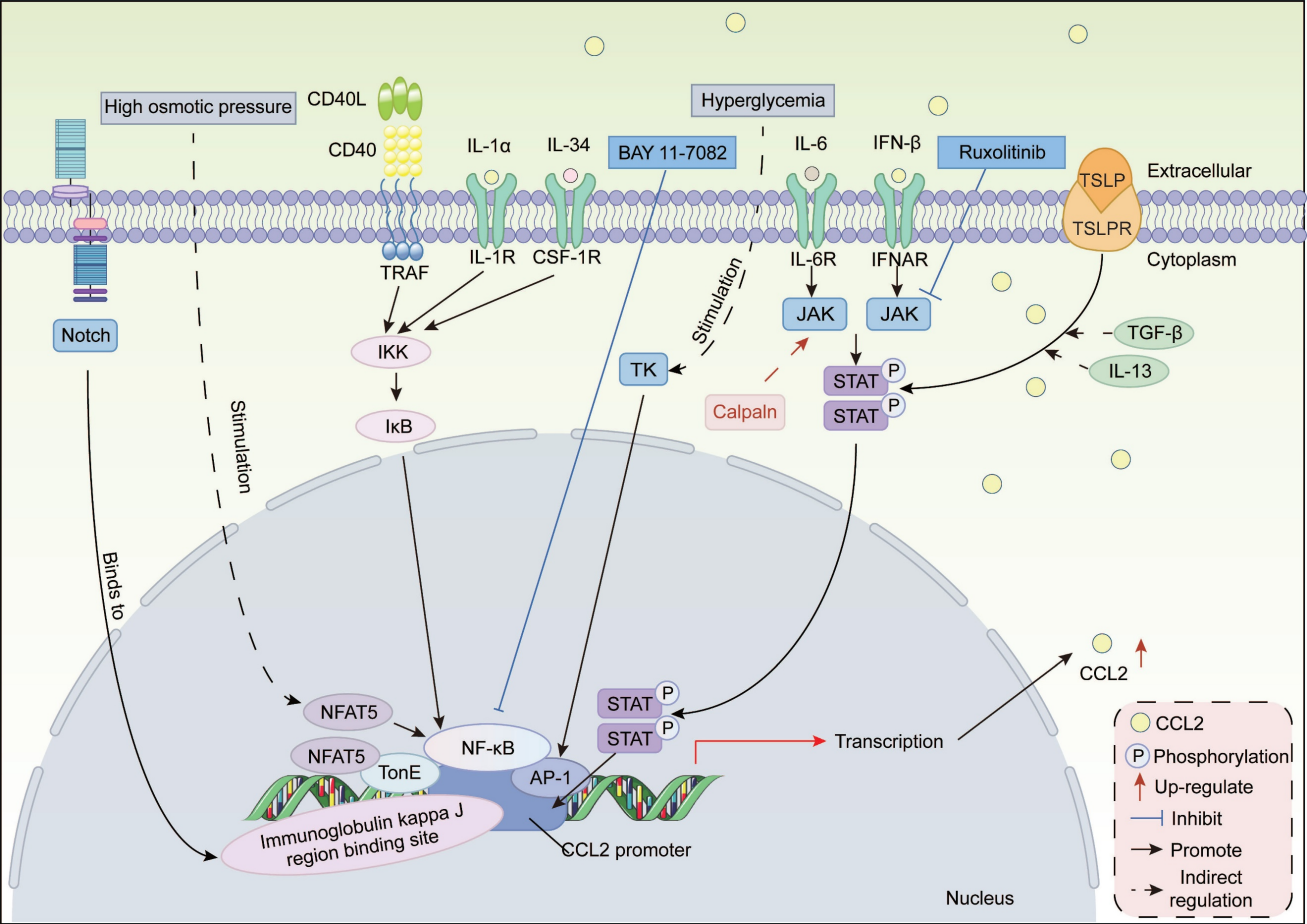
** Upstream regulatory network of CCR2 signaling in fibrosis.** The specific transcription activator NFAT regulates gene transcription by directly binding to the CCL2 gene promoter. Signal-sensing transcription activators enter the nucleus after undergoing modifications such as phosphorylation in response to extracellular signals like hormones, growth factors, and stress, where they bind to specific DNA sequences to regulate CCL2 gene transcription. NF-κB and AP-1 serve as core transcription factors for the CCL2 gene, jointly binding adjacent sites on the CCL2 promoter to synergistically enhance CCL2 transcription. The JAK/STAT pathway and Notch pathway also exert significant transcriptional regulatory roles. These transcription factors often exhibit synergistic interactions and mutual regulation. AP-1, activator protein 1; CSF-1R, colony-stimulating factor 1 receptor; IFN-β, interferon-β; IKK, inhibitor of κB kinase; IL-1α, interleukin-1 α; IκB, inhibitor of κB; JAK, Janus kinase; NFAT5, activator nuclear factor of activated T-cells; NF-κB, p65 subunit of nuclear factor-κB; STAT, signal transducer and activator of transcription; TGF-β, transforming growth factor-β; TK, tyrosine kinase; TRAF, TNF receptor-associated factor; TSLP, thymic stromal lymphopoietin.

In terms of transcriptional regulation, we mainly discuss specific transcription activators and signal-sensing transcription activators **(Figure 1)**. The specific transcription activator nuclear factor of activated T-cells (NFAT) regulates gene transcription by directly binding to target gene promoters or forming synergistic complexes with other transcription factors 89-91. NFAT5 is a widely expressed transcription factor whose activity is regulated by extracellular tonicity 92. Evidence suggests that NFAT5 stimulates CCL2 expression through two distinct mechanisms. First, NFAT5 activates CCL2 transcription by directly binding to TonE, a cis-acting element located upstream of the CCL2 transcription start site 93. Secondly, high osmotic pressure activates NFAT5 in mesothelial cells, which interacts with the p65 subunit of nuclear factor-κB (NF-κB) to synergistically upregulate CCL2 expression, thereby promoting peritoneal fibrosis during continuous ambulatory peritoneal dialysis 75, 94. NFATc3 expression is elevated in the lung tissue and lung macrophages of mice induced with bleomycin (BLM)-induced pulmonary fibrosis. NFATc3 promotes pulmonary fibrosis progression by regulating the expression of CCL2 and CXCL2 genes in macrophages 95.

Signal-induced transcription activators sense extracellular signals such as hormones, growth factors, or stress, undergo modifications such as phosphorylation, and then enter the nucleus to bind to specific DNA sequences, thereby activating or inhibiting CCL2 gene transcription. Common types include NF-κB, activating protein-1 (AP-1), signal transducer and activator of transcription (STAT), and Notch.

##### NF-κB/AP-1

The CCL2 promoter region contains adjacent and evolutionarily conserved NF-κB and AP-1 binding sites, whose synergistic binding drives CCL2 transcriptional expression in pulmonary fibrosis 96, 97. Point mutations or deletions in these binding sites significantly reduce CCL2 promoter activity, thereby impairing normal CCL2 transcription 97. Notably, NF-κB and AP-1 serve as a common integrator pathway for multiple pro-fibrotic signals. CD40 enhances CCL2 secretion in activated human hepatic stellate cells (HSCs) by activating NF-κB 98. Similarly, IL-34 enhances CCL2 expression by activating the NF-κB pathway, thereby promoting macrophage recruitment and polarization, exacerbating cardiac remodeling after myocardial ischemia-reperfusion (I/R) injury 99. In an *in vitro* model of surgery-induced fibrosis in total knee arthroplasty, fibroblasts exacerbate joint fibrosis via the IL-1α/NF-κB/CCL2 signaling pathway 100. Moreover, in peritoneal mesothelial cells, hyperglycemia stimulates CCL2 expression through the tyrosine kinase/AP-1 pathway 101. The cross-organ conservation of this mechanism indicates that the NF-κB/AP-1 complex serves as a common pathway for various stromal cells, including fibroblasts and HSCs, to sense injury and initiate CCL2 expression. BAY 11-7082, an NF-κB inhibitor, reduces CCL2 expression by inhibiting NF-κB p65 activation in a rat myocardial I/R model, thereby decreasing infarct area and late-stage fibrosis 102. This further confirms that CCL2 expression during fibrosis depends on transcriptional regulation by NF-κB and AP-1.

##### STAT family

Members of the STAT family, such as STAT1, STAT3, and STAT6, exhibit functional similarities across different fibrotic contexts. STAT family enhance CCL2 transcription efficiency by forming complexes with the CCL2 promoter or other transcription factors PU.1 and CEBPα 103. In early intestinal inflammation, cells expressing Ly6C^high^ enhance the expression of CCL2 and CCR2 genes via activating the JAK/STAT1 signaling pathway, which is associated with the pathogenesis, exacerbation, and chronicity of acute colitis 104. In systemic sclerosis (SSc) mice, STAT6 deficiency leads to significant downregulation of CCL2 105. In hepatitis C virus (HCV)-induced liver fibrosis, the virus downregulates microRNA-449a (miRNA-449a) and miRNA-107 in the liver, thereby releasing inhibition of the IL-6/JAK1/STAT3 pathway. Activated STAT3 forms a transcriptional activation complex with PU.1 and CEBPα, which synergistically binds to the promoter and activates CCL2 expression 103. Furthermore, in the context of high cholesterol and chronic myocardial ischemia, calpain increases collagen expression by enhancing JAK/STAT/CCL2 signaling, thereby promoting cardiac fibrosis 106. Specifically, The JAK inhibitor Ruxolitinib can inhibit CCL2 transcription by blocking IFN-β-stimulated STAT1 phosphorylation in bone marrow-derived macrophages 107.

##### Notch pathway

The Notch signaling pathway is crucial for multicellular organisms, programmatically controlling cell fate and tissue differentiation during early development 108. An evolutionarily conserved Notch/RBP-J binding site on the CCL2 promoter enables Notch signaling to directly activate CCL2 transcription 109. In non-alcoholic steatohepatitis (NASH) mice, hepatocytes upregulate CCL2 via this site, further promoting MDMs infiltration of into the liver and advancing hepatic fibrosis 109. Bone marrow-specific Notch activation promotes CCR2^+^ macrophage infiltration by upregulating CCL2 expression, ultimately exacerbating renal fibrosis. In addition, Brandt *et al.* utilized chimeric mice lacking Notch3 in hematopoietic cells and/or resident tissue cells to confirm that the development of renal fibrosis and inflammation following unilateral ureteral obstruction (UUO) is significantly associated with upregulation of CCL2 levels. And CCL2 upregulation is Notch3-dependent 110.

It is worth noting that the transcriptional regulatory network of CCL2 in a fibrotic context is extremely complex, with often synergistic and mutually regulatory interactions between different types of transcription factors. Compared to healthy individuals, in the late stages of oral submucous fibrosis, the expression of transcription factor genes cyclic AMP response element-binding protein (CREB), NF-κB, and NFAT5 is upregulated, synergistically promoting CCL2 expression 111. Moreover, thymic stromal lymphopoietin (TSLP) is upregulated in the skin of SSc patients. And the TSLP-TSLPR-STAT3 signaling axis synergistically promotes CCL2 expression in fibroblasts by interacting with pro-fibrotic cytokines TGF-β and IL-13 112. Furthermore, STAT3 is essential for TSLP-induced CCL2 expression 112.

In summary, these transcription activators promote fibrosis progression by binding to specific sites on the CCL2 promoter to activate CCL2 gene expression. Future integration of multi-omics data and disease model information will comprehensively reveal the CCL2 transcriptional interaction network, providing innovative therapeutic strategies for fibrotic diseases.

#### 3.2.2 Epigenetic modifications of CCL2

**Figure 2:**
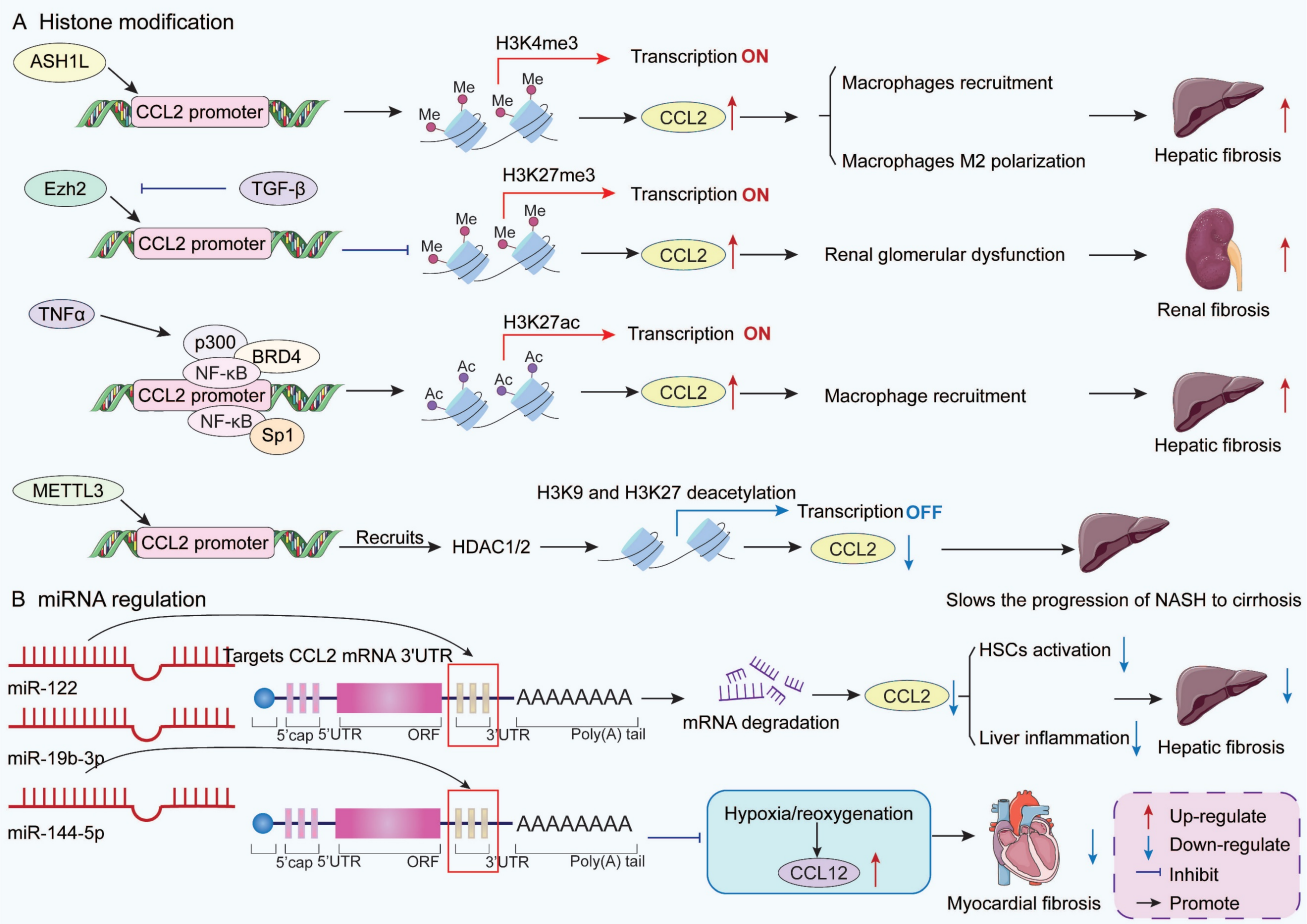
** A summary of the epigenetic mechanisms regulating CCL2 gene expression.** Histone modifications, including methylation, acetylation, and deacetylation, regulate the transcriptional opening and closing of CCL2, thereby influencing CCR2 signaling-mediated cellular activities during fibrosis, thus ultimately altering the fibrotic process. miRNAs regulate post-transcriptional expression by binding to CCL2 and CCL12 to degrade mRNA or inhibit translation. ASH1L, ASH1-like histone lysine methyltransferase; BRD4, bromodomain-containing protein 4; Ezh2, enhancer of Zeste homolog 2; H3K4me3, Histone H3 lysine 27 trimethylation; HDAC1/2, histone deacetylases 1 and 2; HSCs, hepatic stellate cells; METTL3, methyltransferase-like 3; NASH, non-alcoholic steatohepatitis; NF-κB, p65 subunit of nuclear factor-κB; Sp1, specific protein 1; TGF-β, transforming growth factor-β; TNF-α, tumor necrosis factor α; 3'UTRs, 3' untranslated regions.

Some epigenetic mechanisms, such as histone modifications and miRNA regulation, influence CCL2 gene expression at the genetic information level 113 **(Figure 2).** Histone methylation is a type of chromatin modification. Different modification sites and degrees may affect gene transcriptional activation or silencing. ASH1-like histone lysine methyltransferase (ASH1L), a methyltransferase, is highly expressed in activated HSCs and hepatocellular carcinoma cells. Mechanistically, ASH1L significantly upregulates CCL2 transcriptional expression through directly binding to the CCL2 promoter region and catalyzing histone H3 lysine 27 trimethylation (H3K4me3) modification 114. This epigenetic regulatory mechanism promotes the recruitment and polarization of M2-like macrophages, forming an immunosuppressive tumor microenvironment, ultimately accelerating liver fibrosis and hepatocellular carcinoma progression 114. In contrast, in rat renal mesangial cells, TGF-β suppresses CCL2 expression by downregulating enhancer of Zeste homolog 2 (Ezh2) to inhibit H3K27me3 at the CCL2 gene promoter 115. Increased CCL2 expression is associated with fibrosis and glomerular dysfunction in diabetic nephropathy (DN) 115.

Histone acetylation is another important epigenetic mechanism. During liver injury, TNFα promotes histone acetyltransferase p300 interaction with NF-κB and bromodomain-containing 4 to form a complex in mouse liver sinusoidal ECs 116-118. This complex acetylates H3K27 in the CCL2 enhancer and promoter regions, thereby opening the chromatin structure and enhancing CCL2 gene transcription 117. Subsequently, CCL2 recruits CCR2^+^ monocyte-derived macrophages (MDMs) to the liver, promoting liver fibrosis and portal hypertension 117. In addition, Specific Protein 1 (Sp1), a protein that binds to DNA and activates genes, promotes CCL2 transcriptional activation through regulating histone acetylation in the proximal promoter region of CCL2 gene with NF-κB interaction 119, 120. Research has found that histone deacetylation negatively regulates CCL2 gene transcription 121. Li *et al.* confirmed that methyltransferase-like 3 can directly bind to the CCL2 gene promoter and recruit histone deacetylases to induce histone H3K9 and H3K27 deacetylation in the CCL2 promoter region, thereby inhibiting CCL2 gene transcription in the liver 122. This process protects the body from the progression of NASH. NASH is a critical step in the progression of non-alcoholic fatty liver disease (NAFLD) to cirrhosis 123.

miRNAs are a class of endogenous non-coding small single-stranded RNAs that regulate post-transcriptional expression of target genes by binding to their target genes to degrade mRNA or inhibit translation 124. In patients infected with HCV, CCL2 expression is significantly upregulated and negatively correlated with the abundance of liver miR-12, suggesting that miR-122 may negatively regulate CCL2 125, 126. A dual luciferase gene reporter assay demonstrated that miR-122 downregulates CCL2 expression by binding to complementary sequences in the 3' untranslated regions (3'UTRs) of CCL2 mRNA, thereby alleviating liver inflammation 127. An *in vitro* study found that miR-144-5p directly targets the 3'UTR of CCL12 to inhibit the upregulation of CCL12 and CCR2 levels in H9C2 cells induced by hypoxia/reoxygenation 128. This process effectively reduces cell necrosis and fibrosis. Furthermore, hypoxia specifically inhibits miR-146b, thereby releasing TRAF6 inhibition and inducing CCL2 expression 129. This pathway drives cardiac fibrosis and dysfunction and may lead to heart failure. Lan *et al.* compared the miRNA expression profiles between fibrotic and normal livers and found that miR-19b-3p levels were downregulated in activated HSCs. And miR-19b-3p expression was also downregulated in fibrotic human liver tissue. miR-19b-3p can directly bind to the 3'UTR region of CCR2 mRNA, leading to reduced CCL2 mRNA expression and thereby attenuating HSC activation 130.

In summary, CCL2 expression represents the complex outcome of multi-level epigenetic regulation integrating histone methylation/acetylation modifications and miRNA interference. These mechanisms collectively determine CCL2 expression levels within the tissue injury microenvironment, thereby acting as a key switch that drives the fibrosis process by regulating macrophage infiltration. Targeting these epigenetic regulatory nodes, such as ASH1L, p300, or specific miRNAs, hold promise for developing novel therapeutic strategies against diseases including liver fibrosis, diabetic nephropathy, and cardiac fibrosis.

Although progress has been made in studying regulatory factors such as transcription factors and epigenetic modifiers, the dynamic regulatory mechanisms of the CCR2/CCL2 axis in the context of fibrosis remain largely unknown. For example, the specificity of regulatory factors in different tissue microenvironments, the spatiotemporal expression patterns of modifiers, and their correlation with fibrosis stages remain unclear. A deeper understanding of these upstream regulatory networks could not only help reveal the molecular pathophysiological mechanisms of fibrosis but also provide new insights for developing therapies targeting the CCR2/CCL2 pathway to treat fibrotic diseases.

### 3.3 Downstream pathways and co-regulators of CCR2 in the fibrosis process

**Figure 3:**
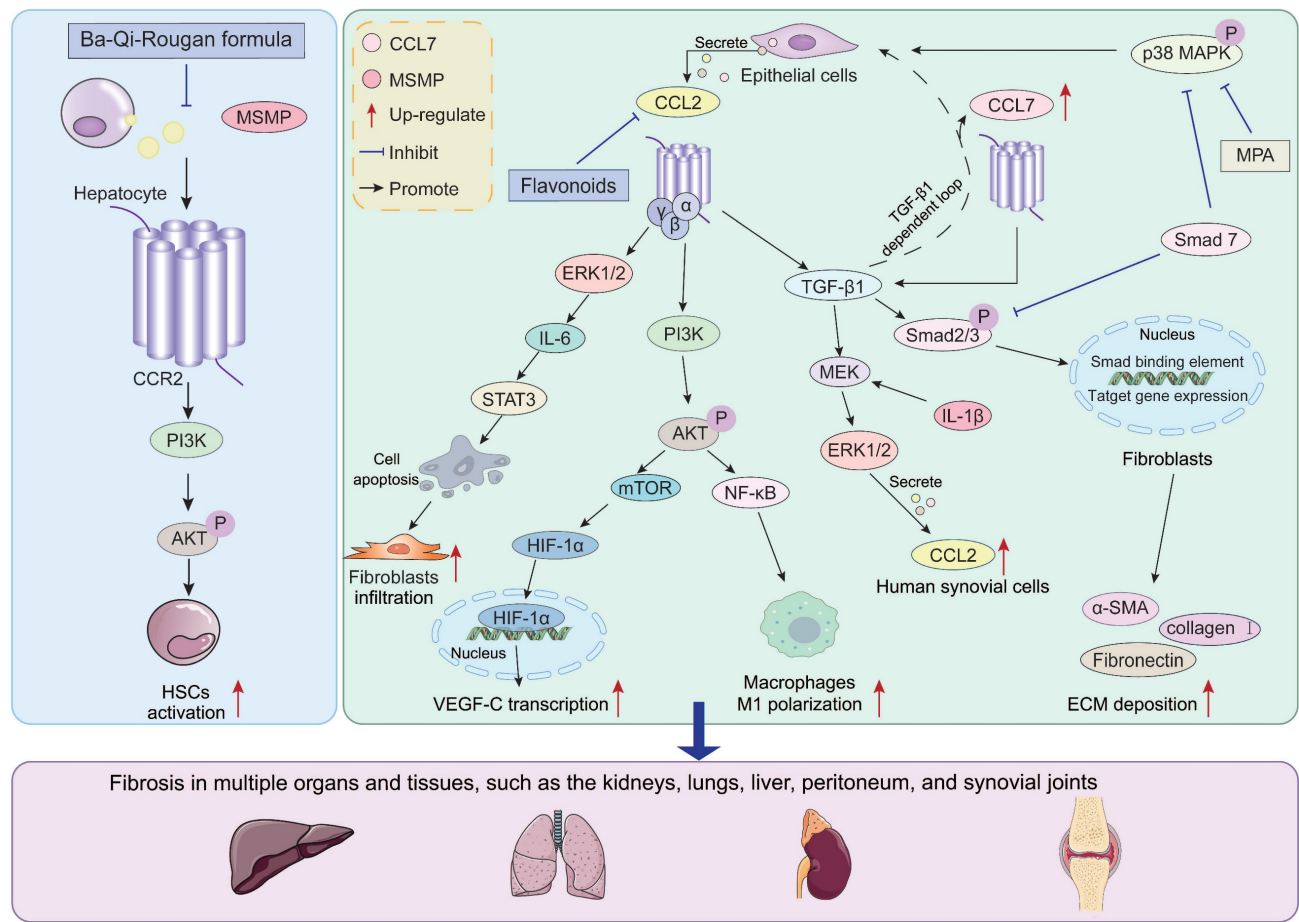
** Downstream pathways and synergistic factors regulated by CCR2 during fibrosis.** CCR2 activates multiple downstream signaling pathways such as PI3K/AKT, TGF-β/Smad, and MAPK by binding to CCL2, CCL7, and MSMP. The activation of these pathways accelerates the fibrosis process. Simultaneously, CCR2 signaling forms a feedback loop with the TGF-β pathway. The TGF-β pathway activated by CCR2 signaling further upregulates CCL2 and CCL7 expression, collectively driving the progression of fibrosis in multiple tissues and organs. AKT, protein kinase B; α-SMA, α-smooth muscle actin; ECM, extracellular matrix; ERK, extracellular signal-regulated kinase; HSCs, hepatic stellate cells; HIF-1α, hypoxia-inducible factor-1α; IL-6, interleukin-6; MEK, mitogen-activated protein kinase kinase; MPA, mycophenolic acid; MSMP, microseminoprotein; mTOR: mammalian target of rapamycin; NF-κB, p65 subunit of nuclear factor-κB; p38 MAPK, p38 mitogen-activated protein kinase; PI3K, phosphoinositide 3-kinase; STAT3, signal transducer and activator of transcription 3; TGF-β, transforming growth factor-β; VEGF-C, vascular endothelial growth factor C.

During the fibrosis process, CCR2 acts as a key regulatory factor, activating multiple downstream signaling pathways through binding with CCL2 and CCL7. The activation of these pathways further upregulates the expression of pro-fibrotic factors. Meanwhile, the CCL2/CCR2 axis synergistically interacts with certain pathways, collectively driving the onset and progression of fibrosis. Here, we briefly discuss important downstream pathways such as PI3K/AKT, TGF-β/Smad, and MAPK **(Figure 3)**.

#### PI3K/AKT

The PI3K/AKT signaling pathway is a key regulator of cell growth, proliferation, and apoptosis 131, 132. Multiple studies have confirmed that activation of the PI3K/AKT pathway is associated with fibroblast activation, epithelial cell damage, and macrophage polarization during fibrosis 133, 134. Notably, the CCL2/CCR2 axis serves as a key upstream signal driving this process. After the CCR2 receptor is activated, its coupled GTP-binding protein βγ subunit (Gβγ) directly binds to the p110 catalytic subunit of PI3K, catalyzing the conversion of phosphatidylinositol 4,5-bisphosphate (PIP2) to phosphatidylinositol 3,4,5-trisphosphate (PIP3), thereby initiating the AKT phosphorylation cascade 135, 136.

Activation of the CCL2/CCR2/PI3K/AKT signaling axis exhibits pro-fibrotic functions across multiple organ fibrosis models. In obstructive nephropathy, the CCL2/CCR2 axis mediates hypoxia-inducible factor-1α (HIF-1α) expression by activating the PI3K/AKT/Mammalian target of rapamycin (mTOR) signaling pathway. Subsequently, HIF-1α drives vascular endothelial growth factor-C expression to regulate UUO-induced renal lymphangiogenesis 137. CCR2-deficient mice exhibit suppressed lymphangiogenesis and reduced renal injury and fibrosis following UUO induction 137. This pathway also serves as a key node regulating immune responses and inflammation, not only promoting M1 polarization of infiltrating macrophages but also mediating hepatic inflammatory responses in NAFLD models, thereby driving liver fibrosis progression 138. Pure total flavonoids from citrus mitigate hepatic inflammation in NAFLD by inhibiting CCL2/CCR2/PI3K/AKT signaling, thereby slowing NAFLD progression to cirrhosis 138. Furthermore, the CCR2/PI3K/AKT signaling axis directly participates in effector cell activation, such as mediating HSCs activation in liver fibrosis 139. Ba-Qi-Rougan formula counteracts liver fibrosis by reducing MSMP expression and inhibiting MSMP-induced HSCs activation via the CCR2/PI3K/AKT pathway 139. Consequently, targeting CCL2/CCR2 and its downstream PI3K/AKT signaling pathway has emerged as a promising strategy for intervening in fibrotic diseases.

#### TGF-β/Smad

The TGF-β/Smad signaling pathway is a core mechanism driving fibrosis. It primarily functions through TGF-β1 binding to the βRII receptor, activating the Smad2/3 complex to translocate into the nucleus, thereby upregulating collagen gene expression and promoting ECM deposition 140-142. In-depth studies reveal a bidirectional feedback loop between this classical pathway and the CCL2/CCR2 axis, with this interaction synergistically amplifying pathological processes in multiple organ fibrosis models. On one hand, CCL2/CCR2 signaling serves as an upstream driver of TGF-β1 expression and functional enhancement. CCL2 not only directly induces TGF-β1 and its receptor TβRII expression in pulmonary fibroblasts but also stimulates collagen synthesis via autocrine or paracrine mechanisms, accelerating fibrosis progression 143, 144. Functional knockout studies further validate this regulatory importance: in CCR2 knockout mice, not only were BLM-induced pulmonary TGF-β1 mRNA levels significantly lower than in wild-type (WT) mice, but fibroblast responsiveness to TGF-β1 stimulation was also impaired due to reduced TβRII and Smad3 expression, resulting in diminished myofibroblast generation 145. Conversely, TGF-β signaling can also induce CCL2 expression in a vicious cycle. For instance, in renal proximal tubule cells, CCL2 expression is directly stimulated by TGF-β1 146. Furthermore, TGF-β and IL-1β synergistically activate the MEK/ERK1/2 pathway, significantly upregulating the expression of CCL2. This is a key mechanism for chronic synovial inflammation and fibrosis 147. Overexpression of the inhibitory Smad7 not only blocks Smad2/3 phosphorylation but also suppresses TGF-β1-induced CCL2 upregulation by downregulating p38 MAPK activation, thereby alleviating peritoneal fibrosis 148, 149.

Notably, this bidirectional regulatory pattern may also apply to other CCR2 ligands. CCL7 expression is upregulated during fibrosis, and CCL7 promotes activation of the TGF-β/Smad3 signaling pathway, thereby increasing type I collagen secretion 150, 151. Concurrently, CCL7 gene expression is stimulated by TGF-β 151. This suggests potential synergistic effects between CCL7 and TGF-β within the fibrotic microenvironment, jointly promoting collagen biosynthesis in fibroblasts.

#### MAPK

The MAPK cascade is a highly conserved signaling pathway that transmits environmental stimuli into the cell nucleus to initiate intracellular responses 152. At least three distinct MAPK families have been identified: p38 MAPK, c-Jun N-terminal kinase (JNK), and ERK.

p38 MAPK primarily functions as a hub for pro-inflammatory and pro-fibrotic signaling, and its activation is crucial for CCL2 production 153, 154. In renal artery stenosis mice, blocking p38 MAPK directly suppressed TNF-α and TGF-β-induced CCL2 upregulation, thereby mitigating renal atrophy and fibrosis 155. Similarly, in peritoneal mesothelial cells, p38 MAPK enhances fibroblast recruitment and infiltration by promoting CCL2 production, thereby driving peritoneal fibrosis 156. Multiple intervention strategies have validated this mechanism. Shenkang injection alleviates diabetic nephropathy by inhibiting p38 MAPK/NF-κB signaling to reduce CCL2/CCR2 activation 157. In rat renal fibroblasts, mycophenolic acid (MPA), an inhibitor of hypophosphimonosulfate dehydrogenase, effectively mitigates renal fibrosis by reducing TNF-α-induced CCL2 expression through downregulating p38 MAPK phosphorylation 158. Collectively, these findings suggest that p38 MAPK activation constitutes a common pathway for CCL2 upregulation and subsequent inflammatory cascades.

In contrast, ERK1/2 primarily functions as a downstream effector activated by the CCL2/CCR2 axis, mediating cell survival and phenotypic maintenance. CCR2 rapidly activates ERK1/2 upon ligand stimulation 78. Activated ERK1/2 promotes fibrosis through two distinct pathways. First, the CCL2/CCR2 axis suppresses fibroblast apoptosis via the ERK1/2/IL-6/STAT3 signaling pathway, contributing to pulmonary fibroblast survival and pulmonary fibrosis development 159. Second, CCL2-enhanced macrophage inflammatory responses correlate with increased ERK1/2 phosphorylation and upregulation of miR-9 expression 160. This differential upstream-downstream relationship suggests that p38 MAPK and ERK play functionally complementary roles in the CCL2/CCR2 signaling network: the former primarily drives CCL2 production and amplification, while the latter mediates CCL2-triggered cellular responses and maintenance.

In summary, during fibrosis progression, the CCL2/CCR2 axis collaborates with downstream cascades including PI3K/AKT, TGF-β/Smad, and MAPK to construct a complex molecular interaction network. The PI3K/AKT pathway, as a core regulator of metabolism and survival, directly activates inflammatory cascades via G proteins, promoting macrophage infiltration and fibroblast activation. The TGF-β/Smad pathway, as the primary executor of matrix deposition, forms a bidirectional positive feedback loop with CCL2/CCR2, directly coupling inflammatory signals to collagen synthesis. Members of the MAPK family assume differentiated yet complementary roles: p38 MAPK, as an upstream hub, is crucial for CCL2 production, forming an autocrine amplification loop, while ERK1/2 acts as a downstream effector mediating cell survival and inflammatory maintenance. The interplay of these three pathways ultimately transforms initial chemotactic signals into persistent tissue remodeling and irreversible fibrotic damage. Future research should further elucidate the spatiotemporal expression patterns of CCR2 within the fibrotic microenvironment and explore combined intervention strategies to achieve more precise anti-fibrotic therapies.

## 4. The role of CCR2-dependent signal transduction in the pathogenesis of fibrosis

### 4.1 Pulmonary fibrosis

**Figure 4:**
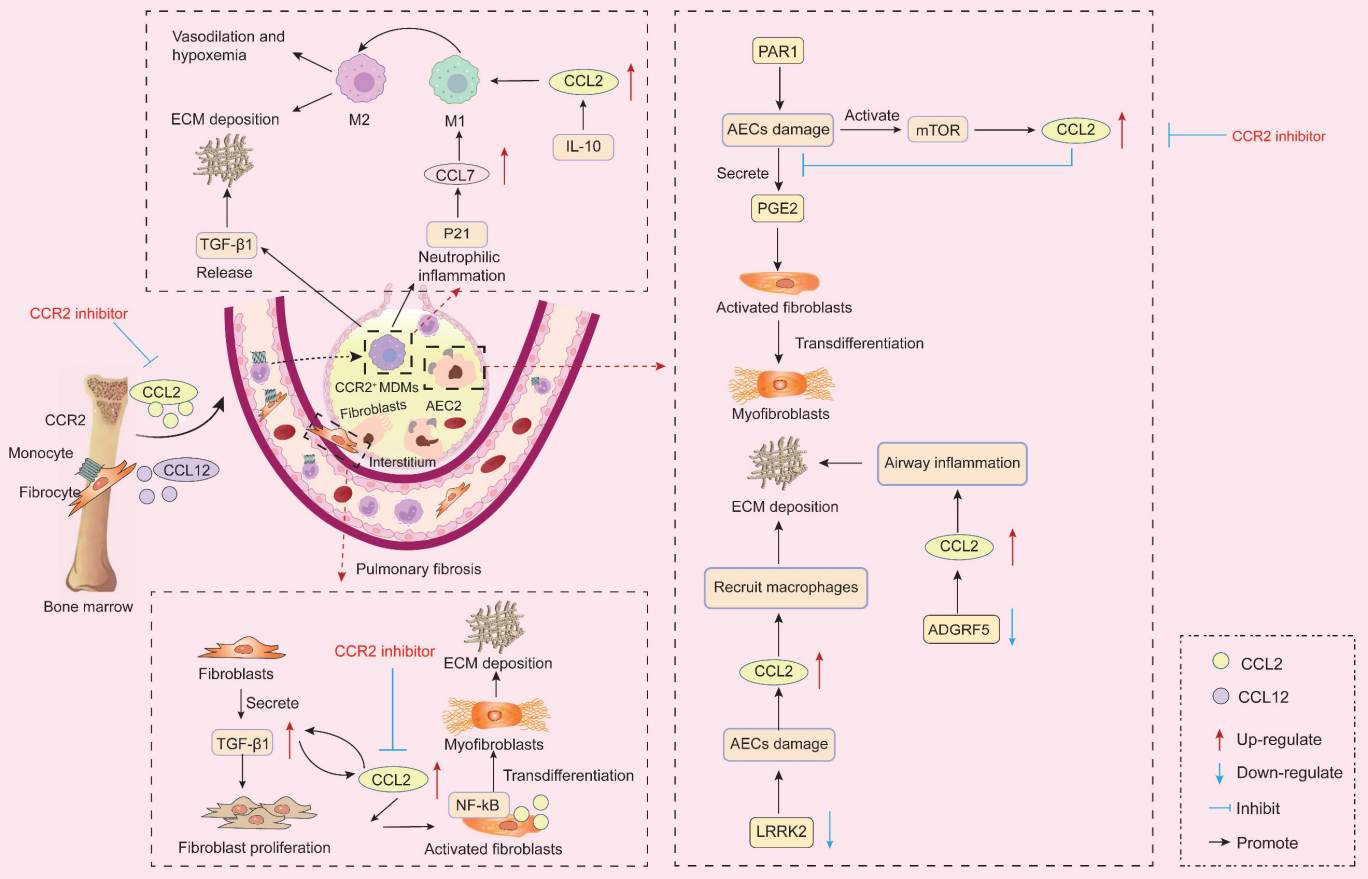
** The role of CCR2-dependent signaling in the pathogenesis of pulmonary fibrosis.** Epithelial cell dysfunction is a key driver of pulmonary fibrosis, with CCR2 initiating damage to AECs through multiple pathways. Furthermore, bone marrow-derived inflammatory monocytes respond to CCL2 and CCL7 chemotactic signals, recruiting CCR2-dependently from the circulation to the lungs. Over time, these cells replace tissue-resident macrophages, differentiating into CCR2^+^ MDMs. CCR2^+^ MDMs highly express inflammatory genes and pro-fibrotic cytokines, driving inflammatory initiation and adverse remodeling. CCR2 signaling not only mediates macrophage polarization, enhancing MDMs infiltration to promote fibrotic progression, but also directly stimulates proliferation of resident pulmonary fibroblasts. Concurrently, bone marrow-derived fibroblasts require CCR2 signaling to be recruited into the alveolar interstitium in response to tissue fibrotic injury. In summary, CCR2 functions as both a regulator of AECs activity and a key factor governing macrophage infiltration and polarization, fibroblast recruitment, and fibroblast activation. AECs, alveolar epithelial cells; ADGRF5, adhesion G-protein coupled receptor F5; CCR2, C-C motif chemokine receptor 2; ECM, extracellular matrix; IL-10, interleukin 10; LRRK2, leucine-rich repeat kinase 2; MDMs, monocyte-derived macrophages; mTOR, mammalian target of rapamycin; NF-κB, p65 subunit of nuclear factor-κB; PAR1, protease-activated receptor-1; PGE2, prostaglandin E2; TGF-β1, transforming growth factor β1.

Pulmonary fibrosis is a chronic, progressive lung disease with a poor prognosis 161. Increasing evidence indicates that IPF is an epithelial cell-driven disease, where abnormally activated alveolar epithelial cells (AECs) produce mediators that promote fibroblast migration, proliferation, and differentiation into active myofibroblasts 162. These processes result in loss of lung elasticity, reduced alveolar surface area for gas exchange, and respiratory dysfunction. Furthermore, the pathogenesis of IPF subtypes differs. Ligand-receptor analysis indicates a monocyte-macrophage chemotactic axis in the myeloid-rich IPF subtype, potentially involving CCL2-CCR2 signaling 163 **(Figure 4)**.

Epithelial cell dysfunction is a key driver of pulmonary fibrosis 164. In the lung, AECs primarily maintain alveolar barrier integrity and represent one of the earliest response mechanisms to lung injury 165. When AECs are injured, compromised epithelial barrier integrity can trigger abnormal fibroblast activation, increased ECM deposition, and structural lung damage. Adhesion G-protein coupled receptor F5 (ADGRF5) is a key regulator of pulmonary surfactant homeostasis in type II alveolar cells. Studies indicate ADGRF5 modulates CCL2 gene expression to maintain immune homeostasis 166. Knockout of ADGRF5 induces airway inflammation mediated by type 2 immune responses and CCL2-induced inflammation 166. In BLM-treated mice, leucine-rich repeat kinase 2 (LRRK2) expression was significantly reduced in type II AECs. Its deficiency caused severe functional impairment in these cells, manifested as impaired autophagy and accelerated cellular senescence. Furthermore, LRRK2-deficient type II AECs exhibited enhanced capacity to recruit pre-fibrotic macrophages via CCL2/CCR2 signaling, leading to progressive pulmonary fibrosis 167. Furthermore, forkhead box F1 (FOXF1) is an endothelial transcription factor involved in pulmonary fibrosis. FOXF1 stimulates Rras transcription, thereby suppressing CCL2 expression. An *in vitro* experiment confirmed that FOXF1-deficient ECs promote pulmonary fibrosis by secreting CCL2 to stimulate macrophage migration and enhance pulmonary fibroblast activation 168. Specifically, under steady-state conditions, AECs suppress fibrosis by inhibiting the conversion of fibroblasts to myofibroblasts through the secretion of the fibroblast inhibitor prostaglandin E2 (PGE2) 169, 170. Studies reveal significantly elevated CCL2 expression in AECs from IPF patients 171. A key pro-fibrotic mechanism of the CCL2/CCR2 interaction is the suppression of PGE2 production in AECs following lung injury, thereby promoting fibroblast-to-myofibroblast conversion and collagen deposition 169. Furthermore, protease-activated receptor-1 activation on AECs may represent a crucial mechanism driving increased local CCL2 release in pulmonary fibrosis 172. Notably, CCR2^-/-^ mice are protected against experimental pulmonary fibrosis. This is because AECs from CCR2^-/-^ mice produce more PGE2 than those from CCR2^+/+^ mice, thereby more effectively suppressing fibroblast proliferation 169. Furthermore, a study revealed that injury in mouse and human primary AECs partially activates the mTOR pathway, leading to increased CCL2 and CCL12 production. These cytokines promote fibrosis through CCR2 activation 15. Targeting the mTOR pathway to reduce CCL2 and CCL12 production in AECs may represent a viable anti-fibrotic strategy.

MDMs are also recognized as key mediators in the pathogenesis of pulmonary fibrosis 173, 174. CCL2 mRNA and protein expression levels in lung epithelial cells from IPF patients are significantly elevated compared to healthy controls, sustaining macrophage recruitment and pulmonary infiltration under pathological conditions 175, 176. Myeloid-derived inflammatory monocytes express CCR2 and recruit from the circulation in a CCR2-dependent manner in response to CCL2 and CCL7, replacing alveolar macrophages and interstitial macrophages over time to ultimately define CCR2^+^ MDMs 177, 178. CCR2^+^ MDMs highly express inflammatory genes and pro-fibrotic cytokines, leading to inflammatory initiation and adverse remodeling 179, 180. In cystic fibrosis mice with chronic inflammation, both inflammatory monocytes and CCR2^+^ MDMs increase in number alongside up-regulated CCL2 expression, while tissue-resident alveolar macrophages decrease 181, 182. Moreover, abundant CCR2^+^MDMs exacerbate fibrosis by driving lung neutrophil-dominant inflammation and TGF-β-dependent pulmonary tissue remodeling 174. Critically, in the cystic fibrosis context, pharmacological inhibition of CCR2 reduces pathological neutrophilic inflammation and TGF-β levels by attenuating MDMs recruitment 174. Similarly, following chemotherapy, bone marrow-derived inflammatory monocytes respond to early fibrotic reactions and migrate to the lungs, where CCR2^+^ MDMs subsequently infiltrate lung tissue, thereby exacerbating radiation-induced pulmonary fibrosis 173. Moreover, Groves *et al.* demonstrated through receptor knockout experiments that mice receiving CCR2-deficient bone marrow showed no pulmonary fibrosis 22 weeks after radiation exposure compared to controls 173. Specifically, pulmonary hypertension (PH) is a fatal disease characterized by progressive pulmonary arteriolar fibrosis and remodeling. Recruitment of CCR2^+^ MDMs leads to pulmonary arteriolar fibrosis correlated with PH severity 183. Specific CCR2 deficiency suppresses CCR2^+^ MDM infiltration, thereby reversing pulmonary arteriolar fibrosis and PH 183. These findings suggest that targeted inhibition of CCR2 may represent a key therapeutic strategy for mitigating pulmonary fibrosis.

Additionally, single-cell RNA sequencing data from IPF patients indicate that the CCL2/CCR2 axis is critical for M1 polarization of macrophages 184. M1 macrophages promote alveolar inflammation and activate myofibroblasts 184. Following radiation or BLM exposure, P21 is upregulated in stressed lung epithelial cells, thereby promoting CCL7 production. CCL7 recruits macrophages by binding to CCR2 and enhances macrophage M1 polarization, ultimately exacerbating lung injury 185. Notably, M2 macrophages also appear implicated in pulmonary fibrosis. Accumulation of M2 macrophages induced by granulocyte-macrophage colony stimulating factor (GM-CSF)/GM-CSFR and CCL2/CCR2 leads to pulmonary fibrosis, promoting vasodilation and hypoxemia, thereby developing hepatopulmonary syndrome 186. Furthermore, IL-10 induces fibrosis through fibroblast recruitment and M2 macrophage activation, a process dependent on the CCL2/CCR2 axis 187. Administration of anti-CCL2 neutralizing antibodies to IL-10-overexpressing mice attenuates pulmonary fibrosis, reducing pulmonary hydroxyproline content and total pulmonary collagen levels 187. The CCL2-mediated M2 macrophage expansion pathway is also present in the lungs of congestive heart failure (CHF) patients, potentially exacerbating pulmonary fibrosis and worsening dyspnea 188.

In summary, CCR2 signaling mediates bone marrow monocyte recruitment to inflammatory tissues, enhances CCR2^+^ MDM infiltration, and ultimately promotes pulmonary fibrosis progression. Notably, Shichino *et al.* demonstrated that CCR2^+^ MDMs exert a protective effect in silica-induced mouse pulmonary fibrosis by inhibiting tissue remodeling-related gene expression, thereby preventing progression from nodular to diffuse fibrosis 189. In distinct experimental models, Liang *et al.* reported that mouse lung-specific overexpression of CCL2 increased MDM infiltration in bronchoalveolar lavage fluid (BALF) and attenuated BLM-induced pulmonary fibrosis in a CCR2-dependent manner 190. These findings suggest that CCR2^+^ MDMs may play variable roles in pulmonary fibrosis progression in a stage- and model-dependent manner. This variability likely correlates with differing activation states in macrophages resulting from variations in fibrotic stimuli and exposure duration 191, 192. Currently, BLM, silica, and fluorescein isothiocyanate (FITC) are commonly used to establish fibrotic animal models. These agents target different pathways and possess distinct half-lives, and the proportions of MDMs and fibroblasts in the lungs of different models vary, potentially leading to differential activation of MDMs.

Additionally, Hadjicharalambous *et al.* documented differentially expressed lncRNAs in human lung fibroblasts following IL-1β activation, demonstrating that MIR3142HG positively regulates IL-8 and CCL2 release. They further established that reduced inflammatory responses in IPF fibroblasts correlate with diminished MIR3142HG expression 193. Furthermore, immunohistochemical analysis of human lung tissue revealed that activated IPF fibroblasts exhibit enhanced contractility and produce abundant CCL2, with the NF-κB signaling pathway participating in CCL2 production and release by these fibroblasts 194. The CCL2/CCR2 axis also upregulates endogenous TGF-β1 expression in pulmonary fibroblasts, enhancing their responsiveness to TGF-β1 and consequently increasing type I collagen levels 143, 145. Moreover, fibroblasts from IPF patients exhibit excessive responsiveness to TGF-β1, IL-13, and CCL2, with these three factors reciprocally reinforcing the fibrotic response 143.

In summary, CCR2 signaling participates throughout the entire process of pulmonary fibrosis. CCR2 serves both as a regulator of AECs function and as a key factor modulating macrophage infiltration, fibroblast recruitment, and fibroblast activation. Beyond basic research, human genetics data also support the critical role of CCR2. Neehus *et al.* reported that homozygous mutations in the CCR2 gene directly cause human progressive polycystic lung disease, characterized by marked peribronchial and parenchymal lymphocytosis with peribronchiolar fibrosis, progressive obstructive airflow limitation, and recurrent secondary infections 195. However, CCR2 signaling may also exert beneficial effects during pulmonary fibrosis progression by influencing specific immune cell subsets. Studies demonstrate increased proportions of the CCR2^+^CD4^+^ T cell subset in BALF from IPF patients, non-IPF pulmonary fibrosis patients, and experimental fibrotic mice 196. This rare T cell subset possesses immunoregulatory functions, capable of suppressing T cell proliferation, alleviating pulmonary inflammation, and inhibiting IPF progression 196. Future intervention strategies targeting CCR2 signaling must comprehensively consider its dual roles in promoting fibrosis and regulating immunity to achieve more precise treatment.

### 4.2 Cardiac fibrosis

**Figure 5:**
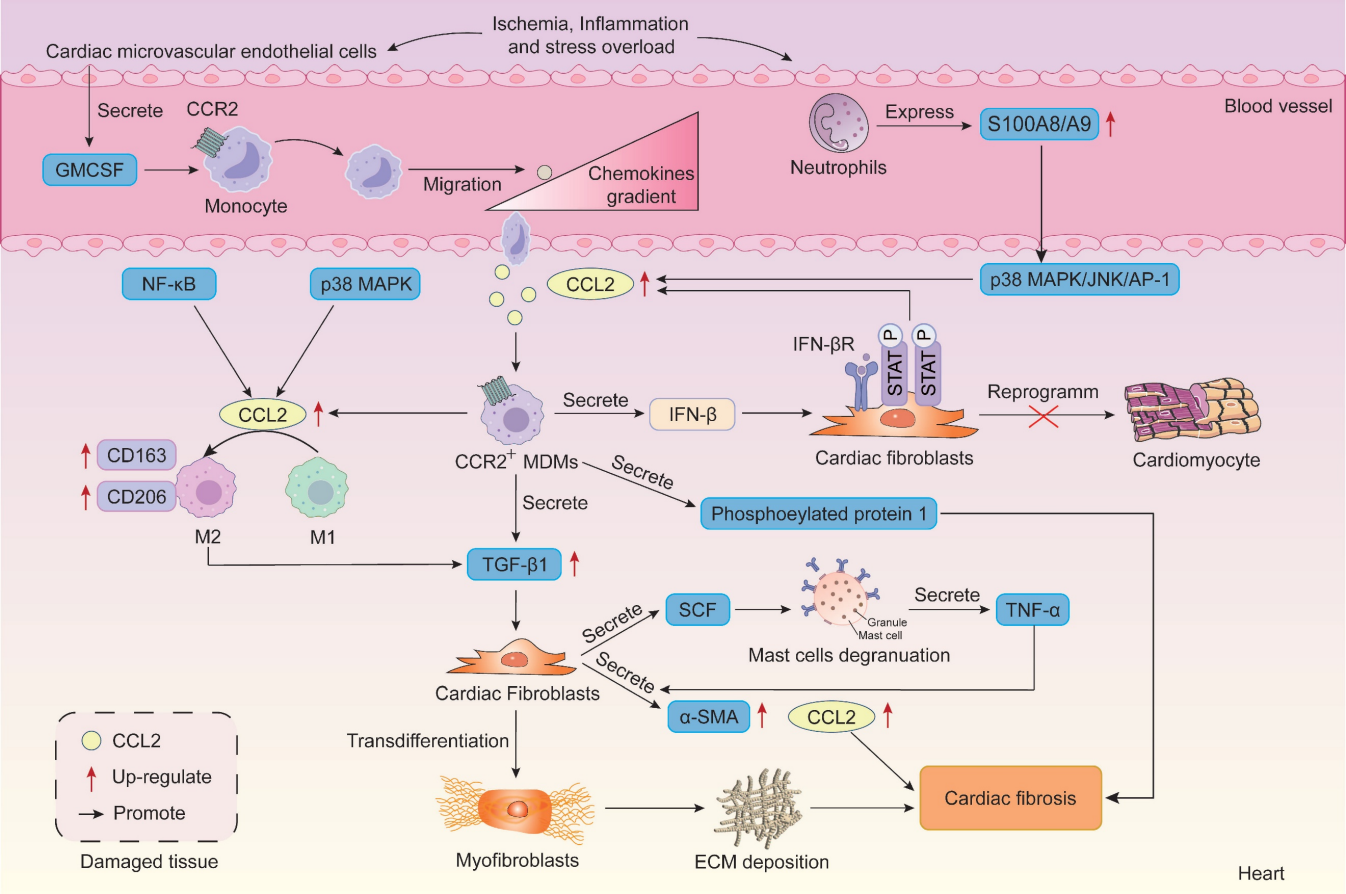
** The role of CCR2-dependent signaling in the pathogenesis of cardiac fibrosis.** During myocardial injury, microvascular endothelial cells release GMCSF to recruit monocytes that differentiate into CCR2^+^ MDMs. These initially exhibit an M1 proinflammatory phenotype and secrete CCL2, which subsequently induces their M2 transformation. The released TGF-β promotes fibroblast-to-myofibroblast conversion, driving fibrosis. At the level of cell-cell interactions, neutrophil S100A8/A9 upregulates CCL2 via the p38 MAPK/JNK/AP-1 pathway, exacerbating macrophage infiltration. Furthermore, mast cell-fibroblast crosstalk and the IFN-β/STAT1/CCL2 positive feedback loop between macrophages and fibroblasts amplify the fibrotic response and suppress cardiac reprogramming. Thus, CCR2 signaling plays a central role in cardiac fibrosis by regulating immune cell recruitment, phenotypic conversion, and multicellular interactions. AP-1, activating protein-1; CCR2, C-C motif chemokine receptor 2; ECM, extracellular matrix; GMCSF, granulocyte-macrophage colony-stimulating factor; IFN-β, interferon-β; JNK, c-Jun N-terminal kinase; MDMs, monocyte-derived macrophages; NF-κB, p65 subunit of nuclear factor-κB; p38 MAPK, p38 mitogen-activated protein kinase; SCF, stem cell factor; α-SMA, α-smooth muscle actin; TGF-β1, transforming growth factor β1; TNF-α, tumor necrosis factor α.

Cardiovascular disease remains a leading cause of morbidity and mortality worldwide. The formation of scar tissue within the heart, known as myocardial fibrosis, represents a terminal feature in nearly all cardiac pathologies. Myocardial fibrosis is characterized by excessive deposition of ECM proteins, which lack the contractile capacity of cardiomyocytes. This leads to cardiac tissue stiffening, reduced compliance, and impaired function, ultimately progressing to heart failure 197, 198. Cardiac diseases such as MI, hypertrophy induced by pressure or volume overload are all associated with progressive cardiac fibrosis 199, 200. Extensive experimental evidence indicates CCL2 mediates cardiac fibrosis in models of ischemic, inflammatory, and stress-induced cardiomyopathies 201, 202** (Figure 5)**.

Studies reveal that recruited MDMs stimulate fibrosis in mouse hearts following aortic arch coarctation surgery. Conversely, resident macrophages suppress cardiac fibrosis. CCR2 expression on macrophages aids classification into resident (CCR2^-^) or circulating-derived (CCR2^+^) types 203. Crucially, the fibrogenic effects of CCL2 primarily stem from the recruitment and activation of CCR2-expressing monocytes and macrophages, leading to production of the pro-fibrotic mediators TGF-β1 and type I collagen 204. CCR2^+^ MDM infiltration is essential for adverse cardiac remodeling during stress overload 200. Early interception of CCR2 signaling or selective depletion of proinflammatory Ly6C^high^CCR2^+^ monocytes during stress overload attenuates late-stage pathological left ventricular remodeling, contractile dysfunction, and cardiac fibrosis 200. In TAC-induced hypertrophic mice, TAC-stimulated neutrophils exhibit upregulation of S100A8/A9, which activates the p38 MAPK/JNK/AP-1 pathway to induce IL-1β and CCL2/CCL6 production. These chemokines promote CCR2^+^ macrophage infiltration into the injured heart 205. Furthermore, CCR2^+^ macrophages mediate PAH-induced atrial fibrillation by secreting phosphoprotein 1 and exacerbate right atrial fibrosis 206.

CCR2 signaling also plays a crucial role in determining macrophage phenotype and ultimately fibrotic progression. During the inflammatory phase of early fibrosis, CCL2 exhibits pro-inflammatory effects similar to LPS, promoting M1 polarization of macrophages and driving the progression from valvular inflammation to valvular fibrosis 207. Moreover, CCL2 is a key regulator of macrophage phenotype during MI healing, specifically promoting M1 polarization 208, 209. Further studies indicate that the role of CCL2 in promoting M1 polarization is significantly attenuated when the p38 MAPK pathway and NF-κB pathway are inhibited 208. In MI mice, cardiac CCL2 deficiency markedly reduced infarct size, collagen synthesis, and cardiac fibrosis, correlating with decreased total macrophage and M1 macrophage numbers in the infarct region 208. Notably, during fibrosis progression, the CCL2/CCR2 axis also upregulates expression of M2 markers CD163 and CD206 on macrophages; these markers are highly expressed and correlate with fibrosis severity 49. Specifically, following acute myocardial I/R injury, cardiac microvascular ECs release large amounts of GM-CSF to attract monocytes migrating to the heart. Under GM-CSF induction, monocytes differentiate into macrophages and switch to the pro-inflammatory M1 phenotype, releasing substantial amounts of inflammatory cytokines and CCL2 47, 210. Subsequently, CCL2 converts GM-CSF-induced M1 macrophages to the M2 phenotype. M2 macrophages release TGF-β to promote the transformation of fibroblasts into myofibroblasts, ultimately leading to cardiac fibrosis 47.

In summary, since macrophage functional phenotypes reflect responses to the local microenvironment and distinct temporal courses of inflammation, the role of CCR2 signaling in regulating macrophage polarization during fibrosis exhibits disease-stage specificity. In early stages, CCR2 signaling promotes macrophage skewing toward proinflammatory phenotypes, thereby driving the transition from chronic inflammation to fibrosis 207. In later stages, CCR2 signaling promotes M2 polarization of macrophages to exacerbate fibrosis 49. Therefore, when targeting CCR2 signaling for fibrotic disease treatment, we must fully consider the disease stage and the timing of targeted intervention. However, the traditional M1/M2 macrophage polarization model fails to meet precision medicine standards, hindering translational progress in clinical research. Recent advances in single-cell sequencing technology have facilitated deeper exploration of macrophage heterogeneity and plasticity. This suggests that future research should integrate single-cell transcriptomics and spatial analysis to track transcriptional changes in CCR2^+^ macrophages across different stages of fibrosis. This approach aims to identify novel subpopulation markers and regulatory pathways, thereby providing precise targets for intervention.

Cardiac fibrosis is mediated by cardiac fibroblasts activation, which differentiate into myofibroblasts under injury or stress. Studies reveal that activated CCR2 signaling induces the recruitment of bone marrow-derived fibroblast precursors to the heart, where these cells differentiate into fibroblasts in response to angiotensin II (Ang-II)-induced cardiac fibrosis 211, 212. *In vitro* experiments demonstrate that CCL2, IL-6, and hypoxia directly promote the differentiation of cardiac fibroblasts into myofibroblasts 213. CCL2 gene deficiency results in significantly reduced ability to recruit proinflammatory macrophages and decreased numbers of cardiac fibroblasts and myofibroblasts 214. Specifically, Wen *et al.* intravenously administered nanoparticles containing the CCL2-binding peptide to AMI mice, neutralizing CCL2 to inhibit CCL2-induced myofibroblast differentiation. This resulted in reduced cardiac myofibroblast formation and decreased total collagen content 215. Furthermore, Luo *et al.* emphasized that early MCs-fibroblast crosstalk and the stem cell factor (SCF)/MC/CCL2/monocyte/macrophage axis constitute key mechanisms driving myocardial fibrosis 216. Specifically, fibroblasts trigger MC degranulation and TNF-α secretion by producing SCF. In turn, MC-secreted TNF-α stimulates fibroblasts to increase CCL2, α-SMA, and TGF-β expression, thereby exacerbating myocardial fibrosis 216.

Building upon these studies, we note recent landmark research published in Cell. Although this study did not directly track the presence of brain-derived fibroblasts in the heart, it revealed a novel brain-heart axis immune mechanism. Brain injury can drive fibrosis in distant organs, particularly the heart, by inducing innate immune memory in myeloid cells 217. Specifically, Simats *et al.* found that monocytes/macrophages in the heart persistently exhibit pro-inflammatory alterations within three months after brain injury, ultimately leading to cardiac fibrosis and dysfunction 217. Further mechanistic studies indicate that IL-1β is a key driver of this epigenetic remodeling. Blocking proinflammatory monocyte migration using CCR2 inhibitors significantly improves post-stroke cardiac dysfunction 217. Although studies have demonstrated that signaling molecules such as CCL2 play important roles in regulating fibroblast fate, whether brain-derived fibroblasts or their precursor cells migrate to the heart via the brain-heart axis and promote fibrosis after brain injury requires further validation. Moreover, the identity, origin, and specific role of brain-derived fibroblasts in cardiac fibrosis remain controversial. Future studies should integrate lineage tracing and single-cell multi-omics technologies to systematically evaluate their contribution to cardiac pathological changes following brain injury.

The role of CCR2 signaling in MI is also a current research hotspot. During MI, elevated CCL2 levels recruit monocytes to participate in the inflammatory response, promoting the replacement of necrotic myocardium with granulation tissue 199. This process contributes to the healing of infarcted myocardium. However, as CCL2 levels rise in the infarcted heart, CCL2 also appears to stimulate fibrous tissue deposition in the injured heart, leading to the development of cardiac interstitial fibrosis and inducing cardiac dysfunction 199. Moreover, within the inflammatory microenvironment of MI, macrophages activate the IFN-β-IFNAR-p-STAT1 axis in cardiac fibroblasts (CFs) by secreting IFN-β, thereby stimulating CFs to secrete chemokines such as CCL2. Subsequently, CCL2 recruits more IFN-β-secreting macrophages, further amplifying its own expression, ultimately forming a self-reinforcing positive feedback loop between CFs and macrophages 218. This positive feedback loop inhibits cardiac reprogramming (i.e. The process where CFs directly convert into cardiomyocytes *in vivo* to regenerate cardiac tissue), leading to adverse remodeling of the infarcted heart. Crucially, macrophages profoundly influence the post-MI repair process, thereby determining subsequent pathological remodeling. Following MI in adult rats, the reparative CCR2^-^ cardiac resident macrophages (cRM) subpopulation is significantly depleted. The excessive recruitment of CCR2^+^ cRMs competitively inhibits the proliferation of reparative CCR2^-^ cRMs, leading to myocardial fibrosis and scar formation 219. Ding *et al.* targeted CCR2^-^ cRMs for artificial intervention by developing a functional conductive cardiac patch capable of inducing CCR2^-^ cRM renewal. This modulates the CCR2^-^ cRM/CCR2^+^ cRM balance, fundamentally correcting the abnormal immune microenvironment and offering a novel approach to suppress cardiac fibrosis 219. These findings indicate that CCL2 acts as both a key factor promoting healing and a primary culprit causing scar formation and adverse remodeling in infarcted myocardium during MI. However, some studies suggest that CCL2 overexpression may actually benefit cardiac repair post-MI. Morimoto *et al.* demonstrated in mice with cardiac-specific CCL2 overexpression that local cardiac CCL2 upregulation reduced infarct size and scarring, promoted myocardial IL-6 secretion and neovascularization, thereby preventing post-MI cardiac dysfunction 213. CCL2/CCR2 also promotes cardiac repair in MI mice by activating the JNK/STAT3 pathway to induce cardiomyocyte proliferation, suppress myocardial apoptosis, and enhance post-MI angiogenesis 220.

In summary, CCR2 signaling exerts multifaceted effects in cardiac fibrosis, including promoting macrophage recruitment and activation, modulating macrophage phenotype, and mediating circulating fibroblast recruitment. Furthermore, extensive studies confirm that CCL2 is significantly induced in infarcted myocardium, aiding infarct healing while also inducing adverse remodeling of infarcted myocardium. However, the detrimental role of CCR2 signaling in post-MI cardiac remodeling and repair remains controversial. Future studies urgently require single-cell spatiotemporal analysis, cell-specific interventions, and more refined ligand-receptor functional characterization to elucidate the dual roles of CCR2 signaling across different repair phases and cell subpopulations, and to explore targeted cardiac intervention strategies.

### 4.3 Liver fibrosis

**Figure 6:**
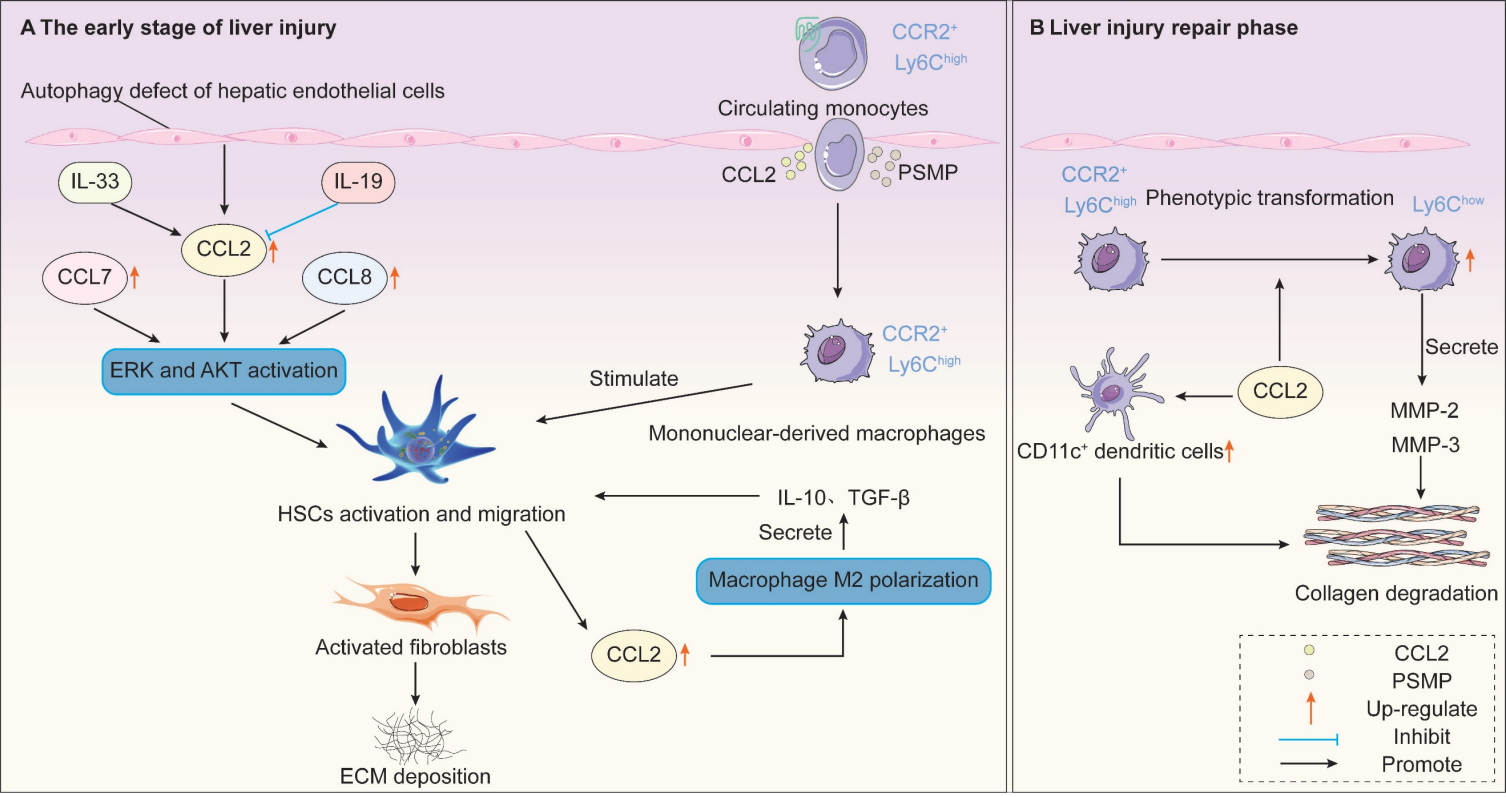
** The role of CCR2-dependent signaling in the pathogenesis of liver fibrosis.** CCR2 signaling exhibits dual roles in promoting fibrosis and facilitating regression across different stages of liver fibrosis. A. In the early phase of liver injury, endothelial cell autophagy defects upregulate CCL2, enhancing HSCs migration via the ERK/AKT pathway while recruiting CCR2-expressing Ly6C^high^ inflammatory monocytes to infiltrate the liver, directly stimulating HSCs activation. Activated HSCs further recruit macrophages via the CCL2/CCR2 pathway and induce their M2 polarization. M2 macrophages secrete IL-10 and TGF-β, which in turn sustain HSCs activation, forming a pro-fibrotic amplification loop. IL-33 promotes CCL2 upregulation and HSCs activation, while IL-19 signaling inhibits fibrosis by downregulating CCL2 expression. B. When liver injury ceases and enters the reparative phase, infiltrating macrophages switch to a reparative phenotype, secreting MMPs to degrade the ECM and promote fibrosis regression. This process partially dependent on CCR2 signaling. The increased proportion of CD11c^+^ dendritic cells during regression may synergistically promote fibrosis reversal, and CCR2 deficiency can impair this process. AKT, protein kinase B; CCR2, C-C motif chemokine receptor 2; ECM, extracellular matrix; ERK, extracellular-signal-regulated kinase; HSCs, hepatic stellate cells; IL-10, interleukin-10; MMPs, matrix metalloproteinases; PSMP, PC3-secreted microprotein; TGF-β1, transforming growth factor-β1.

Liver fibrosis represents the healing response of the liver to various chronic insults, such as viral hepatitis, alcoholic liver disease, non-alcoholic steatohepatitis, and autoimmune disorders. It is characterized by the activation and transformation of HSCs into myofibroblasts, leading to excessive ECM deposition 221-224. Persistent injury can cause severe liver dysfunction, progressing to cirrhosis or even hepatocellular carcinoma. Studies reveal upregulation of CCR2 expression in rodent and human fibrotic livers 225. Extensive research indicates that CCR2 signaling exhibits dual roles in promoting fibrosis and facilitating regression across different stages of hepatic fibrosis **(Figure 6)**. This contradictory function makes it a critical focus for understanding the dynamic regulation of liver fibrosis and developing targeted therapies.

During early and persistent liver injury phases, CCR2 signaling drives fibrosis progression through multiple mechanisms. In the early stages of liver injury, CCR2 is primarily expressed on inflammatory monocytes. Inflammatory monocytes migrate from the bone marrow to the injured liver via CCR2-dependent recruitment. These monocyte-derived CCR2^+^ macrophages accumulate in the periportal regions of patients with NASH and advanced fibrosis 226. Furthermore, these macrophages promote inflammation and angiogenesis while directly stimulating HSC activation 227. In CCR2-deficient mice, impaired recruitment of monocyte subsets results in reduced HSC activation and attenuated liver fibrosis 228. Furthermore, rats administered the human CCL2 mutant 7ND (i.e., with 7 amino acids deleted from the N-terminus of CCL2) via tail vein injection exhibited significantly reduced macrophage infiltration, suppressed HSCs activation, and inhibited liver fibrosis 229. Furthermore, PSMP expression is markedly elevated in human cirrhotic tissues and in mice with experimental liver fibrosis 230. Mechanistically, PSMP promotes inflammatory macrophage infiltration via CCR2 while directly activating HSCs, ultimately exacerbating liver fibrosis 230.

Substantial evidence indicates CCL2 may exert a direct pro-fibrotic effect by stimulating HSCs migration to injured liver tissue. CCL2 has indeed been demonstrated to activate HSCs *in vitro* and stimulate their migration in a dose-dependent manner 231. Nicotinamide adenine dinucleotide phosphate (NADPH) oxidase is a key component in CCR2-mediated HSC activation and chemotaxis during liver fibrosis 232. Following bile duct ligation (BDL), mRNA expression of liver CCR2, CCL2, CCL7, and CCL8 all increased 233. *In vitro* experiments demonstrate that HSCs lacking CCR2 or p47phox (a key component of NADPH oxidase) exhibit impaired ERK and AKT activation, ROS production, and HSCs migration capacity upon stimulation with CCL2, CCL7, and CCL8 233. Among various fibrosis factors derived from bile duct epithelial cells (BECs), CCL2 produced by the innate immune system of the biliary tract is considered most critical in the development of liver fibrosis. CCL2 derived from BECs activates HSCs, thereby promoting periportal fibrosis 234. Furthermore, activating the IL-19 signaling pathway downregulates CCL2 expression in Kupffer cells, thereby reducing HSCs activation and myofibroblast migration to alleviate CCL4-induced liver fibrosis 235. Although HSCs are considered the primary source of type I collagen in fibrotic livers, bone marrow-derived fibroblasts are also implicated in the pathogenesis of liver fibrosis 236. Persistent liver injury triggers the migration of circulating fibroblasts from the bone marrow to the liver, where they differentiate into myofibroblasts. This process is regulated by the CCR2 receptor 237.

When liver injury ceases or enters the repair phase, CCR2 signaling exhibits an opposite, fibrotic regression-promoting function. Studies reveal that CCR2 deficiency reduces inflammatory macrophage migration, leading to diminished HSC activation and ultimately resulting in attenuated liver fibrosis 238. However, once chronic fibrotic injury resolves, the disease regression process is also delayed. This indicates that CCR2 also participates in fibrotic regression. During fibrosis regression, infiltrating macrophages can transition to a reparative phenotype, characterized by downregulated Ly6C expression and secretion of matrix metalloproteinases (MMPs) to degrade the ECM, thereby promoting fibrosis resolution 239, 240. The recruitment and function of these reparative macrophages partially depend on CCR2 signaling. Findings from Mitchell *et al.* support this perspective. In CCR2^-/-^ mice, fibrosis regression was significantly delayed after cessation of CCl₄ injury, accompanied by elevated tissue inhibitor of metalloproteinase 1 (TIMP-1) expression and reduced MMP-2 and MMP-13 expression 238. This suggests that CCR2 deficiency impairs MMPs-mediated ECM degradation, potentially explaining the delayed fibrosis regression. Furthermore, Duffield *et al.* demonstrated that depletion of macrophage populations during injury or during the repair and recovery phases has markedly different effects on the overall fibrotic response 241. Depletion of macrophages during the early injury phase reduces inflammatory responses, diminishes scar formation, and decreases myofibroblast numbers. In contrast, depletion of macrophages during the recovery phase leads to failure of ECM degradation and reduced repair efficiency 241. Furthermore, other cell types may also participate in fibrosis regression. During regression, WT mice livers exhibit increased proportions of CD11c^+^ DCs, which may synergistically promote fibrosis reversal. In CCR2 ^⁻/⁻^ mice, alterations in this cell population further disrupt fibrosis regression kinetics 238.

Furthermore, we note the critical role of the CCR2 signaling pathway in the progression of diseases like viral hepatitis and NASH toward fibrosis. In patients with chronic HCV infection, CCL2 mRNA levels significantly increase in liver tissue as the disease advances 242. Interaction between HCV core protein and gC1qR on macrophages may induce CCL2 secretion via the NF-κB signaling pathway 243. Moreover, hepatic CCL2 mRNA levels correlate directly with histological changes and fibrosis severity 242. In chronic HBV infection, the proportion of monocytes expressing CCR2 increases with disease progression. These cells promote natural killer T cell dysfunction, accelerating the progression from hepatitis to cirrhosis 244. Moreover, in patients with chronic HBV-associated fibrosis, activated HSCs recruit macrophages via the CCL2/CCR2 pathway by upregulating CCL2, inducing their polarization toward the M2 phenotype. M2 macrophages not only exhibit marker expression positively correlated with fibrosis severity but also maintain HSCs activation by secreting cytokines like IL-10 and TGF-β, forming an amplification loop that exacerbates fibrosis 245. Notably, CCL2 also plays a specific role in immune evasion during HBV infection. In advanced cirrhosis caused by chronic HBV infection, persistent liver inflammation may lead to increased spontaneous apoptosis of immune cells, resulting in decreased plasma CCL2 levels 246. This reflects the terminal state of immune dysregulation.

In the pathogenesis of NASH, the CCL2/CCR2 axis plays a complex and seemingly contradictory central role, primarily manifested in recruiting and regulating monocytes/macrophages to influence hepatic inflammation, lipid metabolism, and fibrosis progression. In human NASH liver tissue, enhanced infiltration of CCR2-expressing CD11c^+^CD206^+^ immune cell subsets and increased hepatic CCL2 expression correlate with disease activity 247. In NASH mice, inhibition of CCR2 reduced infiltration of liver CD11b^+^CD11c^+^F4/80^+^ monocytes (i.e., the functional homologs of human CD11c^+^CD206^+^ cells), thereby improving hepatic inflammation and fibrosis 247. Furthermore, a choline-deficient amino acid-defined diet increased hepatic CCL2 expression and CCR2^+^ macrophage infiltration, accompanied by marked hepatic steatosis and fibrosis 248. Additionally, autophagy defects in ECs exacerbated NASH fibrosis by upregulating inflammatory mediators, including CCL2, further underscoring the pivotal role of CCL2 in disease progression 249. Inhibition of IL-33 signaling alleviates liver fibrosis by reducing α-SMA and CCL2 expression, thereby preventing NASH progression to hepatocellular carcinoma 250. However, recent studies reveal that CCR2^+^ macrophage populations may possess important protective functions. Surprisingly, in CCR2 ^-/-^ mice fed a long-term high-fat diet, liver fibrosis significantly increased despite reduced overall macrophage infiltration 251. One explanation is that in CCR2-deficient mice, CX3CR1/CCR2-expressing macrophages fail to appear in the liver, preventing the formation of macrophage aggregates. These macrophage aggregates may exert anti-fibrotic protective effects by clearing dead cells and toxic lipids 251. CCR2 deficiency disrupts the formation of these protective aggregates, leading to accumulation of lipotoxicity and injury signals that instead promote fibrosis progression.

In summary, these findings suggest CCR2 may act as a double-edged sword in hepatic fibrosis. Under persistent injury conditions, CCR2 signaling drives inflammatory monocyte infiltration, amplifying inflammation, activating HSCs, and promoting ECM deposition. However, CCR2 also mediates reparative macrophage phenotype conversion, promotes ECM degradation, and resolves fibrotic scarring. Therefore, if CCR2 inhibitors are used in fibrotic patients, the timing of macrophage infiltration inhibition may be critical to avoid interfering with the recruitment and function of reparative macrophages. We also observed that while CCR2 inhibition has predominantly demonstrated a reduction in liver fibrosis, conflicting results may arise across different experimental models. Consequently, the therapeutic efficacy of CCR2 targeting may be highly dependent on the timing of intervention, the specific disease context, and the resulting balance of macrophage functions. This suggests that future therapeutic strategies must more precisely account for macrophage heterogeneity and their functional roles at different disease stages.

### 4.4 Renal fibrosis

Renal fibrosis represents a common and irreversible pathological pathway in the progression of various chronic kidney diseases to end-stage renal failure. In recent years, CCR2 has been recognized as a key mediator in renal fibrosis, with its expression increasing in multiple chronic kidney diseases such as diabetic nephropathy (DN), autoimmune nephropathy, and ischemic kidney injury 252-254.

The UUO model is a classic model of renal injury that simulates tubulointerstitial fibrosis associated with obstructive nephropathy. In UUO mouse kidneys, tubulointerstitial injury and progressive fibrosis correlate with elevated CCL2 expression and substantial accumulation of CCR2-expressing interstitial macrophages and T lymphocytes 255. The CCL2/CCR2 axis facilitates monocyte recruitment, driving renal inflammation and fibrosis progression. Inflammatory CCR2^+^ macrophages entering the injured kidney during the early stages of tissue injury are a key cause of the subsequent fibrosis observed in UUO 256. Inhibition of CCR2 significantly reduced macrophage infiltration and fibrosis in UUO mice 257. Furthermore, studies revealed that overexpression of human Ang I in UUO mouse tubules reduced CCL2-activated macrophage migration and attenuated late-stage endothelial cell apoptosis, microvascular rarefaction, and fibrosis 258. In contrast to Ang I, Ang II induces CCL2 expression in ECs, where CCL2 subsequently promotes macrophage infiltration and increases endothelial cell apoptosis 258. This process negatively impacts survival rates in chronic kidney disease (CKD) patients. The CCL2/CCR2 axis also serves as a primary pathway for Twist1-mediated renal fibrosis in UUO mice. Increased Twist1 expression in macrophages significantly correlates with the severity of renal fibrosis in UUO mice 259. Knocking out Twist1 partially inhibits CCL2-mediated macrophage chemotaxis and M2 macrophage polarization, thereby delaying the progression of renal fibrosis 259. Furthermore, IL-15 has been demonstrated to reduce CCL2 expression in UUO mice, alleviating fibrosis and decreasing the likelihood of progression to CKD 260. The role of CCR2 in renal fibrosis development is further evidenced by its recruitment of bone marrow-derived fibroblasts into the kidney, promoting the transformation of bone marrow-derived myofibroblasts and the expression of α-SMA and ECM proteins 261. CCR2 deficiency disrupts fibroblast migration to the kidney in response to obstructive injury 262. Specifically, Gonzalez *et al.* demonstrated that CCL7 acts as a direct pro-fibrotic cytokine for renal tubular cells, directly stimulating expression of TGF-β1 downstream mediators CTGF and type I collagen 263. Furthermore, the CCL7/CCR2 axis exhibits dual roles in the progression of tubulointerstitial fibrosis, detrimental in early stages but beneficial in later stages. CCL7 promotes early inflammatory cell infiltration and ECM formation 263. Regulatory T cells (Tregs), the most potent anti-inflammatory cells, contribute to terminating renal inflammation. In later stages, CCL7 maintains protective inflammation by limiting Treg recruitment, thereby attenuating tubulointerstitial fibrosis 263.

Diabetic nephropathy (DN) is a common microvascular complication in long-term diabetic patients, with fibrosis being one of its primary pathological changes. CCR2-mediated monocyte/macrophage recruitment also constitutes a major mechanism of DN-associated renal tissue injury 252. CCR2 is further expressed in cells beyond monocytes, such as podocytes 264, 265. Podocytes constitute a critical component of the glomerular filtration barrier and play a pivotal role in the pathophysiology of DN. Increased podocyte foot process width alongside reduced podocyte number and density directly correlates with elevated urinary albumin excretion rates and progressive renal injury in DN 266, 267. *In vitro* studies revealed that CCL2 binding to CCR2 enhances podocyte chemotaxis with moderate effects on proliferation 268. In type 1 diabetic mice, increased CCR2 expression in podocytes elevated renal fibrillin and type I collagen mRNA expression without enhancing renal macrophage infiltration 264. Notably, the interaction between CCL2/CCR2 signaling and TGF-β1 may exacerbate podocyte apoptosis under diabetic conditions, leading to proteinuria 269. Inhibition of CCR2 protects kidneys from diabetic injury in type 1 diabetic mice 269. CCR2 expression is also increased in podocytes of DN patients 265. In human podocytes, CCL2 binding to CCR2 significantly reduces renin levels via a Rho-dependent mechanism 265. Renin downregulation is closely associated with the development of diabetic proteinuria 270. These findings indicate a pathogenic role for the CCL2/CCR2 axis in DN progression, suggesting that targeting this pathway may represent a novel therapeutic strategy for improving DN outcomes.

Renal fibrosis is also a critical process in autoimmune nephropathies. Urinary CCL2 serves as a non-invasive biomarker for disease activity and treatment response in lupus nephritis patients 271. In lupus nephritis-affected mice, the CCL2/CCR2 axis promotes glomerular macrophage infiltration and induces glomerular microvascular cell proliferation 271. Furthermore, IL-22 binding to IL-22R on renal epithelial cells upregulates CCL2 via STAT3 signaling, thereby promoting macrophage infiltration and exacerbating lupus nephritis 272. A prospective cohort study of 100 patients with autoimmune glomerulonephritis demonstrated that elevated levels of CCL2, sC5b-9, and TGF-β1 correlate with disease activity and poor outcomes 273. Furthermore, CD68-positive interstitial cell infiltration accompanied by CCL2/CCR2 expression represents the most significant prognostic indicator for end-stage renal failure in idiopathic membranous nephropathy 274.

Renal fibrosis is also a key pathological feature in the progression from acute kidney injury (AKI) to CKD. Studies have demonstrated that sustained expression of GM-CSF by renal tubular cells in unilaterally I/R mice, sustained tubular cell expression of GM-CSF significantly increased CCL2 expression by macrophages during the transition from normal renal repair to adverse fibrosis. This led to increased infiltration of CCR2-positive immune cells, including macrophages, DCs, and cytotoxic T cells, into the renal interstitium, thereby promoting persistent tubular injury and interstitial fibrosis during the transition from AKI to CKD 254. The gut microbiota-derived metabolite trimethylamine N-oxide (TMAO) also influences CCR2-mediated renal fibrosis. Choline-rich diets elevate TMAO levels by altering gut microbiota. TMAO promotes sustained high expression of CCR2 in injured kidneys, enhancing macrophage recruitment and infiltration, thereby exacerbating AKI following renal I/R injury and subsequent CKD progression 275. In addition to its role in the aforementioned kidney diseases, Wilkening *et al.* analyzed increased expression of both CCR2 and CCL2 in human focal segmental glomerulosclerosis and demonstrated that infiltration of CCR2-expressing macrophages exacerbates renal injury and fibrotic remodeling 276. Moreover, high sodium chloride intake upregulates CCL2 expression in 5/6 nephrectomized mice, where CCL2 subsequently recruits macrophages to the injured kidney, exacerbating renal fibrosis 277.

In summary, CCR2 signaling serves as a pivotal link connecting renal injury, inflammation, and fibrosis by recruiting and regulating distinct cellular subpopulations. The CCL2/CCR2 axis primarily mediates the infiltration of pro-inflammatory Ly6C^high^ monocytes/macrophages into the kidney, thereby driving inflammatory responses, myofibroblast transdifferentiation, and ECM deposition. However, the biological effects of CCR2 signaling extend far beyond simple pro-fibrotic action. By interacting with CCL7, CCR2 signaling exhibits bidirectional regulatory effects across different stages of renal fibrosis. CCL7/CCR2 signaling drives injury in the early phase, while potentially promoting inflammation resolution and attenuating fibrosis in later stages by limiting Treg recruitment. It is crucial to emphasize that macrophages recruited by CCL2/CCR2 signaling during renal fibrosis are not a homogeneous population. Their function is highly dependent on specific subpopulations. Traditionally, macrophage subpopulations are distinguished by differential Ly6C expression. Ly6C^high^ macrophages, derived from circulating monocytes, exhibit proinflammatory functions and have been identified as the primary injury-driven population in multiple organ fibroses via CCR2 signaling 200, 228, 278. In contrast, Ly6C^low^ macrophages are typically regarded as tissue-resident macrophages primarily involved in immune regulation and tissue repair 279. However, this paradigm is challenged in the specific context of renal fibrosis. Reports indicate that the Ly6C^low^ macrophage subpopulation exhibits a pro-fibrotic phenotype in I/R-induced renal injury mice 280. Yang *et al.* demonstrated that while CCR2 deficiency suppresses inflammatory Ly6C^high^ macrophages recruitment and improves AKI. Due to the compensatory replenishment of Ly6C^low^ macrophages in the kidneys during the chronic phase and their presentation of a pro-fibrotic phenotype, ischemia-induced renal fibrosis still worsens in CCR2-deficient mice 281. This challenges the traditional reparative role of Ly6C^low^ macrophages, revealing the complexity and context-dependent nature of macrophage function in renal fibrosis progression. It also suggests that simply blocking CCR2 signaling globally may disrupt macrophage homeostasis and exacerbate fibrosis. Therefore, future therapeutic strategies require more precise targeting of specific signaling pathways or cell subpopulations at particular stages to achieve optimal therapeutic outcomes.

### 4.5 SSc

SSc is a rare autoimmune disease primarily characterized by fibrosis of the skin and internal organs, leading to diverse clinical symptoms and complications. Studies have shown that CCL2 levels in SSc patients are significantly higher than in controls, with a marked decrease following PGE1 treatment 282. Importantly, the CCR2/CCL2 axis plays a crucial role in SSc by promoting the infiltration of inflammatory monocytes at lesion sites 283. CCL2 mRNA is expressed on infiltrating monocytes and fibroblasts within SSc lesion skin 284. Circulating CD14^+^ monocytes from some SSc patients show upregulation of the ECM component versican and CCL2. Interestingly, versican forms CCL2 chemotactic gradients that attract circulating monocytes and T cells to versican-rich site reservoirs, where these cells subsequently produce additional versican 285. This positive feedback loop involving versican, CCL2, and monocyte influx significantly accelerates SSc progression 285. Intraperitoneal injection of the CCL2 antagonist SKL-2841 reduces inflammatory monocyte and polymorphonuclear cell infiltration while markedly suppressing BLM-induced scleroderma fibrosis 286. This suggests that blocking CCL2 may represent a novel strategy for controlling SSc progression.

One study reported that cultured SSc fibroblasts exhibited significantly elevated levels of spontaneously expressed CCL2 compared to fibroblasts from control skin 287. Interestingly, exogenous administration of CCL2 stimulated autocrine induction of CCL2 mRNA. Thus, increased responsiveness of sclerotic fibroblasts to CCL2 may perpetuate the fibrotic response. Moreover, CCL2/CCR2 may stimulate a subset of quiescent fibroblasts to differentiate into metabolically active myofibroblasts via an autocrine mechanism in early stages of SSc 288. Cross-species comparisons between human and mouse scleroderma reveal IL-13 and CCL2 as specific targets in SSc 289. CCL2 expression is upregulated in IL-13-stimulated human skin fibroblasts, as well as in skin biopsy specimens from inflammatory SSc patients and sclerotic graft-versus-host disease mice 289. In BLM-induced SSc mice, CCL2 promotes dermal fibrosis by directly upregulating ECM mRNA expression in fibroblasts and indirectly through cytokine-mediated effects released by immune cells recruited to the lesion site 290. In SSc patients, CCL2 also induces infiltrating CD4^+^ T cells to differentiate into Th2 cells, which release large amounts of IL-4 and stimulate IL-4R-expressing fibroblasts to produce excessive ECM 291. In CCL2 knockout mice, BLM treatment resulted in mild skin thickening and reduced collagen accumulation, indicating diminished responsiveness to fibrotic stimuli in the absence of CCL2 292. Tight skin 1 (Tsk-1) mice are a widely used SSc animal model. Immunohistochemistry revealed substantial CCL7 expression in the dermis of Tsk-1 mice 150. Furthermore, in the reported experiment, the promoter of the mouse procollagen α2(I) gene was activated by CCL7 150. This suggests that CCL2 may also promote tissue fibrosis by activating ECM synthesis in fibroblasts, thereby contributing to collagen formation.

### 4.6 Colonic fibrosis

Colonic fibrosis represents the outcome of recurrent episodes of chronic colitis. In colitis-prone mice, both CD30L and CCR2 expression are increased in inflammatory monocytes. CD30L activates circulating monocytes via the CCL2/CCR2 axis and NF-κB pathway, enhancing inflammatory responses 293. As an angiotensin receptor blocker, irbesartan reduces the accumulation of Ly6C^high^CCR2^+^ monocytes and fibroblasts along with type I collagen expression in inflamed colons by inhibiting CCL2 production, ultimately suppressing intestinal fibrosis and tumor development 294. The CCR2 ligand PSMP is expressed in patient colitis and colon tumor tissues and is significantly upregulated in dextran sulfate sodium (DSS)-induced colitis tissues in mice. PSMP chemotaxes Ly6C^high^ monocytes from the circulation to inflamed colonic tissue in a CCR2-dependent manner, exacerbating colitis 295. CCR2 also modulates ECM remodeling by upregulating the expression of TIMP-1 296, 297. During chronic colonic inflammation, CCR2^+^ monocytes migrate to the inflamed colon via the CCL2/CCR2 axis and differentiate into CCR2^+^ fibroblasts. Subsequently, CCR2^+^ fibroblasts infiltrate the colon and promote colonic fibrosis by inhibiting collagen degradation through TIMP-1 production 296.

### 4.7 Duchenne muscular dystrophy (DMD)

Muscle fiber necrosis and fibrosis are hallmark features of DMD, leading to fatal diaphragmatic weakness. The diaphragm of DMD (mdx) mice exhibits significantly increased expression of CCR2 and CCL2. CCR2 signaling mediates the recruitment of inflammatory Ly6C^high^ monocytes and the accumulation of CD11b^high^ MDMs, thereby promoting DMD progression 298. In DMD mice, CCR2 deficiency reduces macrophage infiltration by blocking blood-borne inflammatory monocyte recruitment, accompanied by transient improvement in muscle damage and fibrosis 299. This suggests CCR2 inhibition may offer a novel strategy for DMD management. However, this benefit is lost following expansion and pathogenic activation of muscle-resident macrophages, preventing sustained CCR2-mediated improvement in DMD. Mechanistically, these macrophages may induce diaphragmatic fibrosis in late-stage DMD mice by producing persistently elevated levels of fibroblast growth factor 300.

### 4.8 Others

Chronic pancreatitis (CP) is characterized by progressive, irreversible inflammation and fibrosis. In CP rats, PGE2 promotes disease progression by regulating TNF-α-induced CCL2 synthesis in pancreatic acinar cells, which in turn enhances macrophage infiltration 301. Inhibiting cyclooxygenase COX-2 activity reduces PGE2 levels, thereby slowing the progression of pancreatitis and fibrosis 301. Furthermore, the mutant CCL2 reduces serum CCL2 concentrations and impedes monocyte/macrophage recruitment, effectively suppressing di-n-butyl dichloride-induced experimental chronic pancreatitis and subsequent fibrosis in rats 302.

Oral submucosal fibrosis (OSMF) is a chronic disease with a high malignant transformation rate. Increased CCL2 expression in OSMF patients suggests CCL2 may promote OSMF by recruiting myofibroblasts 111, 303. Experimental autoimmune orchitis (EAO) serves as an animal model for studying chronic testicular inflammation and fibrosis. During EAO, CCL2 expression increases, mediating leukocyte infiltration into the testicular parenchyma and recruiting macrophages, thereby promoting fibrosis in testicular inflammation 304. Prostatic fibrosis leads to lower urinary tract dysfunction. Popovics *et al.* demonstrated in a benign prostatic hyperplasia mouse model that CCR2 deficiency impairs monocyte recruitment and macrophage infiltration, thereby improving urinary dysfunction and prostate fibrosis 305. Furthermore, the CCL2/CCR2 axis directly participates in peritoneal mesothelial cell-associated epithelial-mesenchymal transition and ECM synthesis, inducing peritoneal dialysis (PD)-associated peritoneal fibrosis 306. Elevated CCL8 levels in peritoneal effusions of PD patients correlate with increased risk of PD failure, which subsequently leads to peritoneal fibrosis and dysfunction 307.

In summary, the role of CCR2 signaling in organ-specific fibrosis exhibits remarkable complexity. Although CCR2-dependent signaling is widely recognized as a prominent pro-fibrotic pathway that promotes collagen deposition and tissue fibrosis by regulating the dynamic migration and functional activation of diverse immune cells, this effect is not universal. Taking liver fibrosis as an example, CCR2 signaling not only participates in fibrosis formation but also plays a crucial role in the regression phase through mechanisms such as mediating the transition of Ly6C^high^ monocytes to a pro-reparative phenotype and promoting ECM degradation. This organ specificity and stage dependency represent the deep-rooted causes of current research controversies and translational challenges. Therefore, future studies should focus on developing stage-specific and organ-specific intervention strategies. On the temporal dimension, it is essential to identify the functional transition points of CCR2 signaling across different fibrosis stages and explore dynamic regulatory approaches characterized by early moderate interception and late protective enhancement. In the spatial dimension, the tissue origins and microenvironmental differences of CCR2^+^ cells in organs such as the lung, kidney, and heart should be elucidated to avoid simplistic extrapolation of findings from liver fibrosis. Furthermore, cell-specific delivery systems or ligand-selective antagonists should be employed to achieve fine-tuning rather than complete interception of CCR2 signaling.

## 5. CCR2 and its ligands as biomarkers of fibrosis

Fibrosis is a common pathological core of multiple organ chronic diseases, and dynamic monitoring is crucial for assessing disease progression. Research has found that the expression of CCR2 and CCR2 ligands in fibrotic diseases exhibits specificity, making them potential biomarkers for disease diagnosis and prognosis monitoring.

### 5.1 Pulmonary fibrosis

IPF is a progressive inflammatory lung disease that currently lacks effective molecular biomarkers reflecting disease activity or treatment response. Substantial evidence indicates significantly elevated CCL2 levels in both BALF and serum of IPF patients. High CCL2 levels are predictive of poor prognosis in IPF 308-310. Serum CCL2 levels are associated with macrophage activation, and CCL2 upregulation increases the risk of death in IPF patients 311. Moreover, compared with non-fibrotic children, children with pulmonary fibrosis have significantly elevated CCL2 levels and CCR2^+^ T cells in BALF, which are associated with the severity of interstitial lung disease (ILD) 312. Although CCL2 levels were elevated in BALF and serum of ILD patients compared with healthy volunteers, only IPF patients had significantly higher CCL2 levels in BALF than in serum 308. Therefore, simultaneous measurement of CCL2 levels in BALF and serum may help distinguish IPF from ILD.

Specifically, Brody *et al.* developed a radiotracer, ⁶⁴Cu-DOTA-ECL1i, utilizing positron emission tomography to track CCR2^+^ monocytes and macrophages 313. They discovered that in lung tissue from IPF patients, CCR2^+^ monocytes and interstitial macrophages concentrated in perifibrotic regions 313. Furthermore, increased ⁶⁴Cu-DOTA-ECL1i PET uptake correlated with CCR2^+^ cell infiltration. These findings support imaging CCR2^+^ cells within the fibrogenic niche in IPF, providing a molecular target for disease monitoring and personalized therapy 313. Further studies revealed that in pulmonary fibrosis models, early CCR2-PET uptake signals not only reflect pulmonary inflammatory burden but also independently predict subsequent fibrosis severity and treatment response to anti-fibrotic drugs such as nintedanib 314. To further elucidate the imaging capabilities of this novel PET tracer, Heo *et al.* evaluated its efficacy in imaging CCR2^+^ cells in the context of heart transplantation and MI. They observed strong and specific binding to regions containing CCR2^+^ cells, highlighting its potential for imaging human cardiac injury 315. Additionally, transcriptomic analysis revealed that CCL8 expression in fibroblasts from IPF patients was higher than in controls, and CCL8 was involved in key pathways associated with IPF progression. This suggests that CCL8 may be a candidate biomarker for IPF diagnosis and survival prediction 316.

### 5.2 Liver fibrosis

Bai *et al.* analyzed expression dataset GSE84044 of hepatic fibrosis in the GEO database and identified 10 key genes in the protein-protein interaction network, including CCL2 317. Persistently elevated CCL2 levels are considered critical in triggering liver injury and subsequent fibrosis progression, and may serve as a predictive marker for cirrhosis progression 318. Research has found that CCL2 expression is significantly upregulated in the tissues of patients with active cirrhosis 319. Furthermore, liver CCL2 transcription levels are positively correlated with the degree of liver macrophage activation and patient MELD scores (an indicator of the severity of liver fibrosis 320, 321. Although adult cirrhosis is often caused by alcohol abuse or hepatitis, the etiology in children remains unclear. A study conducted genetic sequencing on 14 children with syndromic cirrhosis and identified a recessive mutation in the FOCAD gene as the causative factor. Zebrafish lacking FOCAD exhibited liver damage accompanied by elevated CCL2 expression, suggesting that targeting the CCL2/CCR2 axis may represent a potential therapeutic approach for pediatric cirrhosis 322. Furthermore, CCL2 levels were higher in patients with severe hepatitis recurrence compared to those with non-severe HCV recurrence 323. Functional -2518 CCL2 promoter polymorphisms appear to influence liver CCL2 expression, making HCV patients more susceptible to severe hepatic inflammation and fibrosis 324. CCL2 rs1024610 and the ATGC haplotype are plausible candidate markers for chronic HCV infection 325. These data indicate that CCL2 possesses good predictive capability for identifying severe HCV infection.

### 5.3 Renal fibrosis

Diabetic nephropathy (DN) is a severe complication of diabetes. CCL2 is considered a marker for early-stage DN. With increasing serum and urinary CCL2 levels, the risk of developing new-onset microalbuminuria continues to rise, representing an early sign of DN 326. Currently, DN is the leading cause of end-stage renal disease (ESRD). In DN patients, elevated plasma CCL2 levels are an independent risk factor for ESRD and have significant clinical value in assessing DN prognosis 327. Studies have also found that elevated serum levels of IL-6, NF-κB, and CCL2 are closely associated with renal injury and poor prognosis in DN patients, and combined detection holds significant value for assessing patient status and prognosis 328.

Elevated early-stage urinary CCL2 levels have been demonstrated to correlate with tubulointerstitial fibrosis 329. A meta-analysis revealed that increased urinary CCL2 levels in 596 chronic kidney disease patients predicted renal fibrosis on biopsy and were associated with accelerated eGFR decline 330. Renal CCL2 mRNA expression and urinary CCL2 concentration correlate with the degree of obstruction in hydronephrosis and subsequent renal injury 331. Furthermore, early urinary CCL2 is associated with the late development of interstitial fibrosis and tubular atrophy in renal allografts^332^. Elevated ratio of CCL2 to creatinine (CCL2: Cr) in early urine may aid in identifying patients with interstitial fibrosis and inflammation following kidney transplantation 333. Furthermore, elevated serum CCL8 expression in patients with CKD is associated with advanced CKD staging, urine protein-to-creatinine ratio, and renal fibrosis 334.

### 5.4 SSc

SSc is an autoimmune disease characterized by vascular lesions and uncontrolled fibrosis of the skin and internal organs 335. Delays in the diagnosis and treatment of SSc may lead to uncontrolled disease progression, underscoring the importance of identifying early diagnostic markers. Bioinformatics screening identified the tissue-specific gene CCL2 as an effective biomarker, providing new insights into the mechanisms of SSc 336. Bioinformatics analysis revealed CCL2 as a common signature gene in both IPF and SSc 337. CCL2 expression is elevated in the sclerotic skin of SSc patients, also increased in serum and BALF. Serum CCL2 levels are particularly high in patients with early diffuse cutaneous disease 338. Another clinical study in SSc patients demonstrated that high levels of serum CCL2 are closely associated with increased skin fibrosis and increased risk of internal organ involvement in patients with SSc 339. High circulating CCL2 levels can predict poorer survival outcomes in SSc patients 340. A recent longitudinal analysis of SSc patients revealed a year-by-year decline in circulating CCL2 levels, accompanied by improvement in skin sclerosis 341. Therefore, CCL2 can serve as a biomarker for assessing disease activity and severity of visceral involvement in SSc 342. Furthermore, elevated serum CCL7 levels in SSc patients are positively correlated with the severity of skin fibrosis and pulmonary fibrosis 343. Serum CCL13 is also specifically elevated in SSc patients 344. However, data from Gambichler *et al.* indicate that CCL13 levels in SSc patients are not significantly different from those in healthy controls 345. Therefore, further research data is needed to confirm whether CCL13 can be used as a biomarker for SSc.

### 5.5 Others

In patients with myeloproliferative neoplasms (MPN), the percentage of CCR2^+^ cells is significantly associated with the severity of myelofibrosis, and CCR2 expression on CD34^+^ cells correlates with high-risk classification of MF and the presence of circulating primitive cells 346. Moreover, single nucleotide polymorphisms (SNPs) in CCL2 have been shown to influence the bone marrow microenvironment in MPN. The CCL2 rs1024611 SNP (i.e., CCL2 gene regulatory region -2518A/G substitution) alters its transcriptional activity, thereby upregulating CCL2 expression levels 347. Analysis of MF patients indicates that homozygous status for the CCL2 -2518G allele is an independent prognostic factor for reduced overall survival 348. Furthermore, the polymorphic allele (G) is more prevalent in patients who progressed to MF from polycythemia vera (PV) or essential thrombocythemia (ET), and its presence correlates with adverse clinical outcomes 347. Collectively, these findings suggest that the CCL2 rs1024611 polymorphism may represent a candidate genetic susceptibility factor for MF and an independent risk factor for PV/ET-to-MF progression 349.

Beyond studies in MPN, the CCL2 -2518A/G polymorphism has demonstrated relevance in other diseases. Research in German SSc patients suggests the G variant may be associated with increased CCL2 gene transcriptional activity 350. Overexpression of the CCL2 -2518 GG homozygote in patients suggests that carrying the CCL2 G allele is a risk factor for SSc, and this polymorphism may influence CCL2 expression in skin fibroblasts of SSc patients 350. However, another study in European Caucasians failed to replicate this finding, reporting no association between this SNP and SSc susceptibility 351. Additionally, the CCL2 -2518A/G polymorphism has been linked to acute pancreatitis, acute recurrent pancreatitis, and chronic pancreatitis 352. Furthermore, serum CCL2 levels were significantly elevated in all patients with pancreatic inflammatory diseases, and the CCL2 -2518G allele was significantly overexpressed in patients with acute recurrent pancreatitis 352. A genetic association study involving 79 Korean male patients with alcoholic chronic pancreatitis and 82 male controls further indicated that CCL2 polymorphisms may correlate with disease severity in alcoholic chronic pancreatitis 353.

In summary, these studies demonstrate that the specific expression levels of serum CCR2 ligands serve as a standard risk assessment and predictive tool for fibrotic diseases, enabling monitoring of disease progression and evaluation of prognosis. Moreover, following fibrotic injury, CCR2 PET imaging detects CCR2⁺ monocytes/macrophages localizing to periportal areas of fibrosis, with the number of CCR2⁺ cells reflecting disease activity and severity. Thus, the potential core value of CCR2 PET imaging lies in its ability to non-invasively localize and quantify CCR2⁺ monocytes/macrophages, thereby capturing spatiotemporal dynamics unattainable through conventional biopsy or blood tests. Therefore, CCR2-PET not only serves as a non-invasive biomarker for monitoring disease activity and progression but also holds promise as a tool for assessing drug efficacy in clinical trials, accelerating the development of anti-fibrotic drugs and the implementation of precision treatment strategies. Consequently, CCR2 and its ligands hold promise as potential biomarkers for fibrotic diseases, offering effective strategies for early diagnosis, dynamic monitoring, and stratified prevention and treatment.

However, translating these biomarkers from basic research to clinical application remains challenging. Circulating chemokine levels do not necessarily correlate with tissue levels, meaning plasma CCL2 concentrations may not accurately reflect the degree of CCL2 activation within the vascular wall. This limitation reduces the sensitivity of biomarkers. Combining CCR2 ligands with other clinical diagnostic parameters may enhance diagnostic effect. Furthermore, the metabolic networks within the body are highly complex, and disease manifestations exhibit significant heterogeneity. Selected biomarkers may show variations across different individuals, disease stages, or treatment interventions, thus limiting their clinical applicability. Therefore, future efforts should validate the feasibility of these biomarkers in larger, more representative clinical sample populations.

## 6. Targeting CCR2 signaling as a therapeutic strategy for fibrotic diseases

Extensive research has confirmed that CCR2 signaling is a key therapeutic target for treating various organ fibrotic diseases. The crucial role of CCR2 in the pathogenesis of fibrosis has driven the development of various treatment strategies, including small molecule antagonists, natural Chinese herbal preparations, gene therapy, and mesenchymal stem cell (MSC) transplantation **(Table 2)**. These therapeutic strategies focus on inhibiting the activation of the CCL2/CCR2 axis and have demonstrated significant potential in preclinical studies and clinical trials. Among these, cenicriviroc (CVC) and NOX-E36 have entered the clinical trial phase **(Table 3)**.

### 6.1 Small molecule antagonists

#### CVC

CVC is a novel oral dual-target CCR2/CCR5 antagonist with good safety and bioavailability 354, 355. The anti-inflammatory and anti-fibrotic effects of CVC have been confirmed in many fibrotic animal models 356-358. CVC has been demonstrated to improve hepatic inflammation and fibrosis by inhibiting inflammatory monocyte recruitment and reducing macrophage infiltration 225, 359. *In vitro* studies revealed that CVC suppresses transcription of key pre-fibrotic genes in macrophages by inactivating the STAT1/NF-κB/ERK signaling pathway 225. CVC also suppressed CCL2-induced increases in hepatic fatty acid synthase and adipose differentiation-related protein while enhancing acyl-coA oxidase 1 and proliferator-activated receptor gamma coactivator-α expression, suggesting a mechanism for mitigating hepatic steatosis 360. CVC also reverses hepatic steatosis, insulin resistance, inflammation, and fibrosis in NASH mice by inhibiting macrophage M2 polarization 361, 362. Furthermore, in acute sclerosing cholangitis mice treated with the apoptosis inhibitor BV6, CVC therapy alleviates liver damage and bile duct fibrosis 357. CVC also reduces macrophage infiltration and disease severity in DMD mice 363.

Currently, clinical studies using CVC for the treatment of NASH-related liver fibrosis have made significant progress, and CVC is expected to become a commonly used drug for the clinical treatment of liver fibrosis. CVC has demonstrated good tolerability and safety in over 1,100 trial participants, including patients with cirrhosis and mild to severe liver dysfunction 355, 364. In a Phase IIb clinical trial (NCT02217475), Friedman *et al.* found that one year of CVC treatment resulted in significant improvement in liver fibrosis, and no worsening of steatohepatitis in NASH patients 365. One year later, their research team further reported that CVC exhibits sustained antifibrotic effects, with greater efficacy in patients with advanced fibrosis, particularly those with stage 3 fibrosis 365. CVC is currently undergoing a Phase III clinical trial (NCT03028740) to evaluate and confirm the efficacy and safety of CVC treatment for adult NASH 366. Results showed that while CVC is safe and well-tolerated in patients, its primary endpoint—fibrosis regression (22.3% in the CVC group vs. 25.5% in the placebo group) and no worsening of NASH (23.0% in the CVC group vs. 27.2% in the placebo group)—was not met 367. Mechanistically, CVC selectively targets CCR2/CCR5 to block liver infiltration by Ly6C^high^ proinflammatory MDMs, demonstrating clear efficacy in inhibiting the initiation of fibrotic signaling 359. However, the failure of this Phase III trial highlights the challenges of targeting CCR2 during the fibrosis reversal phase.

As previously noted, the regression of liver fibrosis relies not only on suppressing inflammation but also requires the participation of reparative macrophages. Studies confirm that in the late stages of fibrosis, CCR2 also participates in recruiting reparative macrophages with pro-degradative functions. These cells promote ECM degradation by producing MMPs, serving as key drivers of active fibrosis reversal. Long-term CVC blockade, while suppressing pro-inflammatory monocyte infiltration, may also disrupt the recruitment of this reparative population, thereby undermining the intrinsic capacity for fibrosis regression. Further single-cell transcriptomics studies reveal that hepatic macrophages exhibit heterogeneity and adapt their phenotypes in response to microenvironmental signals within fatty liver, enabling compensatory interactions among distinct macrophage subpopulations 368. Inhibiting the CCR2⁺ subset alone is insufficient to eliminate pro-fibrotic populations like TREM2⁺ scar-associated macrophages, making it difficult to drive active reversal of deposited collagen 369. Moreover, Phase III trial data show that the CVC group even had a lower rate of fatty liver inflammation resolution than the placebo group, clearly demonstrating that anti-inflammatory strategies alone struggle to achieve histological reversal under sustained metabolic stress.

In summary, anti-fibrotic treatment requires precise timing control to avoid excessive blockade of the CCR2 receptor during the inflammatory suppression process, as this could impede the critical pathways for initiating tissue repair. Concurrently, future anti-fibrotic drug development should shift toward mechanism-complementary combination therapy strategies to achieve multidimensional interventions encompassing anti-inflammatory effects, metabolic regulation, and pro-regenerative repair. Recent studies reveal synergistic effects when combining CCR2 inhibitors with FGF21 agonists in alleviating steatohepatitis and fibrosis 370. CVC inhibition blocks hepatic infiltration of inflammatory monocytes, while FGF21 agonists improve obesity-related metabolic dysfunction, confirming the therapeutic potential of integrating these approaches in NASH patients 370.

#### RS-504393

Research has shown that the small-molecule CCR2 antagonists RS-102895 and RS-504393 exhibit significant anti-inflammatory and anti-fibrotic effects 254. The use of RS-102895 to block CCL2/CCR2 signaling can reduce renal fibrosis in UUO mice and renal vascular hypertension mice 257, 371. Furthermore, treatment with RS504393 significantly reduced urinary albumin excretion and mesangial expansion while inhibiting the synthesis of pro-fibrotic and pro-inflammatory cytokines, thereby restoring renal function in type 2 diabetic mice 372. In BLM-induced scleroderma mice, RS-504393 effectively reduces the levels of TGF-β1 and type I collagen in the skin 373. Intraperitoneal injection of RS-504393 reduces valvular inflammation and fibrosis in rheumatic heart disease rats 207. RS-504393 also blocks macrophage infiltration and bladder fibrosis in rats with bladder outlet obstruction 374. However, RS-504393 remains in the preclinical development stage, and future clinical data are needed to assess its safety and efficacy.

#### NOX-E36

As a CCL2 antagonist, NOX-E36 specifically binds to and inhibits CCL2, demonstrating potent anti-fibrotic effects in DN animal models by restoring glomerular endothelial glycocalyx and barrier function 375, 376. Furthermore, NOX-E36 protects DN mice from developing diffuse glomerulosclerosis 377. NOX-E36 also accelerates the resolution of toxic and metabolic liver fibrosis in two experimental models by suppressing the early influx of Ly6C^high^ monocytes, thereby shifting the hepatic macrophage balance toward the reparative Ly6C^low^ subpopulation 378, 379. A Phase IIa study involving 75 patients with type 2 diabetes and proteinuria showed that NOX-E36 is safe, well-tolerated, and has renal protective effects.

#### Others

INCB334 is an efficient and orally bioavailable CCR2 antagonist with strong inhibitory activity against human CCR2 but moderate activity against mouse CCR2 380, 381. Inhibition of macrophage accumulation using INCB3344 prevents Ang II-induced vascular fibrosis and blood pressure elevation 382. McIntosh *et al.* reported a novel CCR2-targeted drug, OPL-CCL2-LPM, comprised of the human CCL2 fused to a truncated form of the enzymatically active A1 domain of Shigella dysenteriae holotoxin 383. Data from a rat model of anti-thymocyte serum-induced mesangioproliferative glomerulonephritis indicate that treatment with OPL-CCL2-LPM leads to CCR2^+^ MDMs depletion and reduces mesangial cell proliferation and ECM synthesis 383. Therefore, treatment with LPM may slow down downstream fibrotic events occurring in many glomerulonephritis and nephrotic syndromes. Further clinical trials are needed to validate the safety, specificity, and efficacy of LPM.

In addition, long-term treatment with anti-CCL2 monoclonal neutralizing antibodies can inhibit macrophage accumulation, fibroblast proliferation, and TGF-β expression, thereby effectively alleviating myocardial fibrosis 202. Moreover, this neutralizing antibody alleviated myocardial fibrosis without reducing myocardial hypertrophy, and improved diastolic dysfunction without affecting blood pressure or systolic function. Carlumab is a specific CCL2-inhibiting immunoglobulin G1κ monoclonal antibody 384. However, in Phase II clinical trials, carlumab failed to demonstrate clinical benefit in IPF patients and even suggested a trend toward worsening prognosis in some dose groups 385. The reason for this failure may be that compensatory mechanisms became overly activated under CCL2 inhibition. However, the failure of this therapy stems not from a single mechanism but from multiple factors, including pharmacokinetic limitations and patient population heterogeneity. From a pharmacodynamic perspective, carlumab may fail to achieve sustained and adequate CCL2 neutralization due to insufficient dosage, inadequate administration frequency, or antibody affinity limitations, resulting in incomplete target inhibition. Regarding patient heterogeneity, the tendency toward worsened prognosis in the medium-dose group (5 mg·kg⁻¹) may correlate with poorer baseline characteristics. Patients in this group were older, more obese, had lower baseline forced vital capacity and 6-minute walk distances, and exhibited higher rates of oral corticosteroid use 385. This suggests that patients with higher disease severity or metabolic inflammation may exhibit poorer responses to this therapy, potentially even experiencing paradoxical worsening. In summary, although carlumab failed to demonstrate clinical benefit, the therapeutic value of targeting the CCL2/CCR2 axis remains intact. Developing next-generation interventions with optimized pharmacokinetic profiles or mechanisms that circumvent compensatory pathways retains research potential.

### 6.2 Natural Chinese herbal preparations

Many herbal medicine formulations have been shown to have good anti-fibrotic effects 386, 387. Honokiol is a polyphenolic compound isolated from the bark of magnolia officinalis. This compound reduces renal interstitial fibrosis in UUO rats by inhibiting CCL2 expression 388. Tianhuang formula improves liver fibrosis by inhibiting the CCL2/CCR2/MAPK/NF-κB signaling pathway 389. Quercetin is a natural flavonoid compound with anti-inflammatory and cardioprotective effects. Quercetin downregulates CCL2 expression via the ERK1/2-C/EBPβ pathway, significantly improving cardiac inflammation and fibrosis in autoimmune myocarditis mice 390. Dachaihu decoction has been shown to reduce CCL2 levels in the pancreas, thereby decreasing macrophage infiltration and fibrosis associated with chronic pancreatitis 387. RNA sequencing combined with network pharmacology studies suggest that Fu-Gan-Wan exhibits potential in alleviating carbon tetrachloride-induced hepatic fibrosis, lipid peroxidation, and iron metabolism disorders in mice 391. This effect is mediated by the NF-κB/CCL2/CCR2 and nuclear factor erythroid 2-related factor 2/heme oxygenase-1 (Nrf2/HMOX1) pathways 391. Moreover, Puerarin exhibits strong anti-inflammatory effects by inhibiting the NF-κB signaling pathway and reducing CCL2 and CCL7 production in colon tissue 392, 393. Puerarin also prevents myocardial fibrosis after MI by reducing CCL2 expression and inhibiting the TGF-β1 pathway 394. Arctigenin downregulates CCL2 expression by inhibiting the ROS/ERK1/2/NF-κB pathway, ultimately reversing tubular EMT in the renal interstitial fibrosis process 395, 396.

Astragalus exhibits anti-inflammatory and anti-fibrotic effects in various diseases. Astragalus alleviates peritoneal fibrosis in PD rats by inhibiting CCL2 and TGF-β1 pathway 397. Furthermore, the antifibrotic effects of astragalus saponin IV (AS-IV) have been confirmed in various animal models of cardiovascular diseases 398, 399. AS-IV alleviates renal injury in streptozotocin-induced DN rats by inhibiting CCL2 expression mediated by NF-κB 400. Activated pancreatic stellate cells (PSCs) play a key role in the pathogenesis of pancreatic fibrosis and inflammation. The polyphenolic compound curcumin inhibits the activation of AP-1 induced by IL-1β and TNF-α, thereby suppressing CCL2 production and ultimately inhibiting PSC activation 401. Curcumin also reduces CCL2 secretion by inhibiting Kupffer cell activation, thereby reducing Ly6C^high^ monocyte infiltration to prevent carbon tetrachloride-induced liver fibrosis 402, 403. Curcumin can also effectively limit the progression of fibrosis in experimental fatty liver mice 404. In brief, the evidence for the antifibrotic effects of these herbal formulations remains at the preclinical stage, and future clinical trials are needed to explore their toxicological characteristics, human safety thresholds, and clinical adverse reactions.

### 6.3 Gene therapy

Anti-CCL2 or CCR2 gene therapy can effectively reduce fibrosis, as demonstrated in various fibrotic animal models such as UUO mice and DMD mice 137, 257, 299. Kitagawa *et al.* demonstrated that CCR2 gene knockout reduces UUO-induced renal interstitial fibrosis in mice 257. Furuichi *et al.* also obtained similar results using a renal I/R model, with CCR2-deficient mice exhibiting resistance to renal injury and fibrosis 405. Moreover, CCL2-deficient mice show reduced collagen fiber formation and are protected from BLM-induced skin fibrosis 292. Tian *et al.* established a new strategy based on CCR2 small interfering RNA silencing (siCCR2) by loading multivalent siCCR2 with tetrahedron framework DNA nanostructure vehicle (tFNA-siCCR2) to alleviate liver fibrosis 406. tFNA-siCCR2 can restore the immune cell landscape and establish an anti-fibrotic microenvironment by inhibiting the accumulation of macrophages and neutrophils in mouse livers 406. Chen *et al.* synthesized FNA-siCCR2 targeting M1 macrophages to block macrophage accumulation in lung parenchyma, thereby improving chemoradiation-induced pulmonary fibrosis in mice 184.

Moreover, the truncated [1+9-76] CCL2 analogue (also known as 7ND) has been shown to be a weak inhibitor of CCL2/CCR2 signaling in mice 407. In BLM-induced pulmonary fibrosis mouse lung tissue, 7ND mediates anti-fibrotic effects in mouse fibroblasts by reducing CCL2 and ECM protein levels 407. Mesenchymal stem cells transduced with 7ND significantly mitigate BLM-induced lung injury 408. 7ND also prevents liver fibrosis in rats by blocking macrophage infiltration and inhibiting HSCs activation 229. Currently, the method of using mutant gene transfection to antagonize CCL2/CCR2 signaling has demonstrated good anti-fibrotic effects. Delivery of the N-terminal deletion mutant 7ND of the human CCL2 gene to skeletal muscle significantly improves renal fibrosis in UUO mice by reducing type I collagen deposition and TGF-β expression 229. Furthermore, 7ND gene transfection therapy alleviates dimethylnitrosamine-induced liver fibrosis and interstitial fibrosis following experimental MI 409, 410. 7ND gene transfection therapy can also effectively inhibit fibrosis of the infrapatellar fat pad in arthritis rats 411.

### 6.4 MSC transplantation

MSCs and derived exosomes (EVs) have emerged as promising alternative therapies for treating various organ fibroses due to their potent immunomodulatory capabilities 412, 413. MSCs primarily reduce mature macrophages expressing high levels of CCR2 by inhibiting CCL2 production in fibroblasts and macrophages 413. This finding supports MSC-based clinical treatment for SSc patients. Furthermore, MSC-derived EVs (MSC-EVs) reduce CCL2 production by inhibiting ERK1/2 phosphorylation 412. This leads to decreased monocyte-macrophage recruitment to the lungs, effectively alleviating pulmonary fibrosis 412. MSC-EVs also diminish CCL2 expression, macrophage infiltration, and production of fibrosis markers type I collagen and α-SMA in liver injury 414. Furthermore, human amniotic fluid-derived stem cells therapy can reduce CCL2 expression in BALF from BLM-injured mice, thereby inhibiting collagen deposition and fibrosis progression 415.

In summary, these data support the clinical translation of targeting CCR2 signaling to alleviate fibrosis in patients. To maximize therapeutic efficacy, further clinical trials are needed to provide more beneficial databases to elucidate the efficacy of targeting CCR2.

### 6.5 Systematic comparison of different CCR2/CCL2 axis targeting strategies

Regarding oral bioavailability, small-molecule antagonists represent the only strategy with viable oral administration potential. INCB3344 demonstrated 47% oral bioavailability in mice 380. In contrast, monoclonal antibodies, gene therapies, and MSCs therapies lack oral feasibility and require intravenous or local injection. Notably, oral bioavailability varies significantly among herbal formulations. Most active constituents, such as polysaccharides, flavonoids, and alkaloids, exhibit poor water solubility and generally low absorption rates.

Regarding target specificity and off-target risks, small-molecule antagonists exhibit high target specificity. INCB3344 demonstrates over 100-fold greater selectivity for CCR2 compared to highly homologous receptors such as CCR1 and CCR5. While off-target risks exist, they are generally manageable. Monoclonal antibodies exhibit exceptional target specificity, with anti-CCL2 antibodies demonstrating high antigenic epitope selectivity and low off-target risk. Natural herbal medicines exhibit the weakest targeting specificity. Their inhibition of the CCR2/CCL2 axis is an indirect effect, primarily achieved by modulating multiple signaling pathways including NF-κB, MAPK, and PI3K, with no evidence of direct binding to the target. Consequently, natural compound formulations carry a higher off-target risk. Gene therapy achieves highly specific gene silencing through siRNA sequence design. MSCs exert immunomodulatory effects via multi-targeted, non-specific mechanisms. Its off-target risk is moderate, as its distribution, survival, and differentiation within the body are difficult to precisely control. Allogeneic cells also carry the risk of immune rejection.

Clinical validation of small-molecule antagonists remains limited. CVC has demonstrated good safety and tolerability in patients with liver cirrhosis, with no adverse effects on body weight, liver/kidney weight, or liver function 364. However, in a 24-week study evaluating CVC safety in patients with primary sclerosing cholangitis, 20 participants (83.3%) reported at least one treatment-emergent adverse event, including fatigue, rash, and dizziness, with most events classified as mild or moderate 355. Although no serious adverse events were observed, data on the long-term hepatic and renal burden and drug interactions associated with oral administration of small-molecule antagonists require further experimental investigation. For monoclonal antibodies, the inability to achieve sustained, complete target coverage may paradoxically exacerbate inflammation through compensatory overexpression. Therefore, heightened vigilance is warranted regarding the risks of immunogenicity and compensatory activation with monoclonal antibodies. Traditional Chinese herbal formulations possess extensive historical usage experience but lack robust evidence-based medical support. Some herbs exhibit long-term hepatotoxicity and nephrotoxicity, with safety data insufficient for modern toxicological evaluation. Regarding gene therapy, although anti-CCL2 gene therapy has shown short-term efficacy in liver, kidney and joint fibrosis, its long-term safety has not been systematically evaluated. Mesenchymal stem cells have a low survival rate and poor phenotypic stability in inflammatory microenvironments, and there are potential risks such as tumorigenicity and abnormal differentiation caused by gene-modified cells. Therefore, the long-term safety of mesenchymal cell therapy also needs to be studied.

Small-molecule antagonists are relatively low-cost with well-established chemical synthesis processes. Monoclonal antibodies, however, incur higher costs due to complex production processes and stringent quality control requirements. Natural Chinese herbal medicines have lower costs, but establishing systems for extracting, purifying, and quality controlling active ingredients significantly increases expenses. Gene therapy is extremely costly, primarily due to high expenditures in viral vector production, purification, and quality control. MSCs therapy is also highly expensive, as the autologous cell preparation process is time-consuming and poses significant challenges in quality control.

Comparative analysis reveals that small-molecule antagonists offer a viable therapeutic strategy for fibrotic diseases due to their favorable oral bioavailability. However, their clinical translation faces significant barriers primarily due to target limitations. While monoclonal antibodies exhibit high specificity, the clinical failure of carlumab starkly highlights the systemic limitations of pure ligand-neutralization strategies when confronted with risks of compensatory activation. Traditional Chinese herbal medicines require identification of active components and key targets to serve as precise interventions for fibrosis treatment. Gene therapy and mesenchymal stem cell therapy offer advantages in locally inhibiting CCR2 signaling and mitigating compensatory risks, yet clinical translation remains challenged by delivery efficiency, long-term safety, and high costs.

## 7. Summary and outlook

As a key cytokine, CCR2 is considered a pivotal node in the progression of fibrotic diseases. CCR2-mediated fibrosis involves a series of cellular or molecular signaling pathways, forming a complex regulatory network with multiple levels and pathways. Extensive experimental data support that CCR2 signaling participates in nearly the entire process of fibrosis formation, being closely associated with inflammatory monocyte recruitment, macrophage polarization, fibroblast production and activation, and ECM deposition 208, 288, 290, 295. Notably, CCR2 signaling may exert detrimental or beneficial effects at different stages of fibrosis 238, 263. CCR2 not only promotes early fibrotic injury but also facilitates late-stage fibrosis resolution by enhancing macrophage MMPs secretion for ECM degradation and maintaining immunosuppressive T cell subsets, including Tregs and CCR2^+^CD4^+^ T cells 196, 238, 263. In this context, blocking CCR2 alleviates early fibrotic injury but also delays fibrosis resolution 238-240. This poses challenges for targeting CCR2 signaling in fibrotic disease therapies. We must not only inhibit the capacity of CCR2 signaling to amplify inflammation and fibrotic injury but also preserve matrix degradation and T cell immunosuppression.

Therefore, when targeting CCR2 signaling to treat fibrotic diseases, it is crucial to identify the stage of disease progression and select the optimal timing for targeted intervention. Prioritizing CCR2 antagonists during the fibrogenesis phase has demonstrated efficacy in multiple preclinical studies and clinical trials 365, 373, 379. During the regression phase, combining MMP activators and immunomodulators enables precise regulation. Combined application of CVC and MMP1 has been demonstrated to reverse liver inflammation and fibrosis *in vivo* while reducing adverse reactions 416. Furthermore, adoptive transfer of CD4⁺CD25⁺FoxP3⁺ Tregs alleviates BLM-induced pulmonary fibrosis in mice, accompanied by decreased CCL2 production and reduced circulating fibroblasts accumulation 417.

Currently, clinical trials targeting the CCL2/CCR2 axis have demonstrated promising anti-fibrotic effects, with most participants reporting significant relief of fibrotic symptoms 355, 365. However, the failure of the CVC and carlumab clinical trials profoundly highlights the complex challenges encountered during the translation from basic research to clinical application. Based on the above analysis, we attribute the failures to four primary factors: (1) Limitations in target biology. The Phase III failure of CVC indicates that merely blocking CCR2⁺ monocyte infiltration is insufficient to reverse fibrosis driven by heterogeneous populations such as TREM2⁺ scar-associated macrophages 368, 369. Moreover, excessive blockade of CCR2 signaling may also interfere with the recruitment of reparative macrophages during the late stages of liver fibrosis, thereby impairing the liver own capacity for fibrosis regression. (2) Redundancy and compensatory activation of signaling pathways. This is the primary reason anti-CCR2 therapies prove completely ineffective or even harmful in certain scenarios. The inherent redundancy of chemokine networks makes neutralizing a single ligand highly prone to triggering compensatory responses. For example, in FITC-induced pulmonary fibrosis mice, systemic CCL2 blockade failed to inhibit fibroblast recruitment, attributed to compensatory effects from other CCR2 ligands 418. This aligns with the Phase II clinical trial of the CCL2 neutralizing antibody carlumab, which failed to improve IPF and demonstrated compensatory increases in serum free CCL2 levels 385. Similarly, complete CCL12 knockout failed to protect BLM-injured mice from pulmonary fibrosis, whereas lung epithelial cell-specific CCL12 knockout did 15. One explanation is that complete CCL12 deficiency leads to significant compensatory increases in other CCR2 ligands, such as CCL2 and CCL7, whereas CCL12-specific deletion attenuates this compensatory response while reducing the accumulation of pro-fibrotic macrophages 15. A more specific case comes from Gurczynski *et al.* In the context of γ-herpesvirus infection following bone marrow transplantation, CCR2 deficiency not only failed to confer benefit but exacerbated pulmonary fibrosis 419. The mechanism involves impaired recruitment of classical monocytes due to lost CCR2 signaling, but the body activates an alternative pathway. Massive infiltration of αGR1-resistant MHC II^+^ neutrophils that secrete IL-17, directly promoting fibroblast activation and ECM deposition 420. (3) Limitations of disease models. The anti-fibrotic effects observed in preclinical experiments failed to replicate in humans, exposing the limitations of traditional rodent models in simulating human disease. (4) Patient Heterogeneity. As a highly heterogeneous progressive disease, IPF demonstrated in Phase II clinical trial of carlumab that patients with higher disease severity or metabolic inflammation may respond less favorably to the therapy. This outcome was attributed to the lack of detailed clinical stratification based on underlying conditions and metabolic inflammatory profiles.

The aforementioned clinical translation failures provide crucial insights for CCR2-targeting strategies. First, drug design must address redundancy. Simply neutralizing the CCL2 ligand may fail to achieve sustained, complete signal blockade, potentially exacerbating chemokine-driven processes through feedback activation. Therefore, to maximize therapeutic efficacy, we should consider multi-targeted combined inhibition of CCR2 signaling to circumvent compensatory mechanisms. Combined siRNA targeting Sparc, CCR2, and Smad3 demonstrated favorable anti-fibrotic effects in BLM-induced mice, with suppressed activation of both macrophages and fibroblasts 421. Concurrently, future trials must prioritize precise patient stratification to mitigate impacts on therapeutic efficacy.

In summary, CCR2 signaling serves as a key driver of fibrosis, contributing to the progression of fibrosis in multiple organs and tissues. In the future, gaining a deeper understanding of the spatiotemporal regulatory mechanisms of CCR2 signaling will facilitate the development of more precise therapeutic strategies for fibrosis, enabling effective interventions that shift from inhibiting fibrosis progression to promoting its regression. Specifically, research should focus on the following key directions: (1) Precisely deciphering the functional heterogeneity of CCR2^+^ cells within the fibrotic microenvironment. It is recommended to combine high-throughput, high-dimensional techniques such as single-cell sequencing, spatial transcriptomics, and organoid models to systematically map the molecular characteristics and spatial distribution of CCR2^+^ cells across different stages of fibrosis. This will clarify the dynamic evolutionary relationship between fibrosis-promoting subpopulations (CCR2^+^Ly6C^high^ macrophages) and reparative subpopulations. (2) Explore the potential application of CCR2/CCL2 as biomarkers for patient stratification and personalized therapy. Integrate clinical information with molecular phenotypes to establish a CCR2 axis-based activity-stratification system, providing evidence for guiding targeted drug administration. (3) Develop organ-specific targeted delivery systems to enhance tissue selectivity in CCR2 signaling intervention. Particularly in diseases like liver fibrosis and pulmonary fibrosis, prioritize designing organ-targeted nanocarriers or fusion proteins to achieve efficient local inhibition of fibrosis progression while minimizing systemic side effects such as immunosuppression.

## Figures and Tables

**Table 1:** A summary of MCP family members that bind to CCR2 with high affinity.

CCR2 Ligands	Species specificity	Structural homology with human CCL2	Target cells of chemotactic action	Biological function	Disease association
CCL2/MCP-1	Both humans and mice	68%	T cells, NKs, monocytes, macrophages, neutrophils, B cells, DCs, mast cells, endothelial cells, epithelial cells, microglia, fibroblasts, tumor cells	Inflammatory response, immune regulation, angiogenesis, tissue repair and regeneration, tumor growth and metastasis, fibrosis	Atherosclerosis, hypertension, cancer, diabetes, respiratory tract infection, osteoarthritis, RA, hepatic fibrosis, neurodegenerative diseases
CCL7/MCP-3	Both humans and mice	73%	T cells, NKs, monocytes, macrophages, DCs, eosinophils, neutrophils, basophils, endothelial cells, epithelial cells, fibroblasts, mast cells, astrocytes, stromal cells, tumor cells	Immune regulation, inflammatory responses, antiviral immunity, tissue regeneration	Cancer, allergic diseases, viral infection, cardiovascular disease, diabetes, AKI, ALI, osteoarthritis, neuropathic pain, pneumonia, renal tubulointerstitial fibrosis
CCL8/MCP-2	Human only	69%	T cells, NKs, DCs, monocytes, basophils, macrophages, fibroblasts, endometrial cells, mast cells	Inflammatory responses, Th2 immune response, skeletal muscle regeneration	Cancer, allergic diseases, AIDS, ARDS, graft-versus-host disease, IPF, preeclampsia, viral pneumonias
CCL12/MCP-5	Mice only	66%	Macrophages, T cells, astrocytes, endothelial cells, epithelial cells	Function similar to human CCL2	Cancer, ALI, cardiovascular disease, ICH, IPF, osteoarthritis
CCL13/MCP-4	Human only	65%	T cells, NKs, DCs, monocytes, macrophages, eosinophils, basophils, mast cells, endothelial cells, epithelial cells, fibroblasts, chondrocytes, tumor cells	Function similar to human CCL7/8	Cancer, allergic diseases, RA, cancer, SSc, Alzheimer's disease, cardiovascular disease

Abbreviations: AIDS, acquired immune deficiency syndrome; AKI, acute kidney injury; ALI, acute lung injury; ARDS, acute respiratory distress syndrome; B cells, bursa dependent lymphocytes; CCL2/MCP-1, monocyte chemotactic protein-1; DCs, dendritic cells; ICH, intracerebral hemorrhage; IPF, Idiopathic pulmonary; fibrosis; NKs: natural killer cells; RA: Rheumatoid arthritis; SSc, Systemic sclerosis; T cells, thymus dependent lymphocytes.

**Table 2:** Preclinical studies of drugs targeting CCL2/CCR2 axis for the treatment of fibrotic diseases.

Drug	Target	Experimental model	Method	Result	Refs
CVC	CCR2/CCR5	Mice with liver fibrosis induced by CCl_4_	4 dosages of CVC (15 mg/kg body weight) per week, for 6 weeks, i.p.	Inflammatory FSCN1^+^ macrophages and HERC6^+^neutrophils ⬇, ECM accumulation ⬇, liver fibrosis ⬇	225
		Mice with intrabiliary injection of BV6	15 mg/kg/d, for 5 days, s.c.	Monocyte recruitment ⬇, macrophage accumulation ⬇, liver fibrosis ⬇	357
		Alcohol-fed mice	15 mg/kg/d, for 6 weeks, s.c.	Alcohol-related liver mRNA⬇, TNF-α, IL-1β, IL-6, and CCL2 ⬇, proinflammatory Ly6C^high^ MDMs ⬇	360
		Fibrotic steatohepatitis mice	50mg/kg, two times a day, i.g.	Histological NASH activity and hepatic fibrosis ⬇, Ly6C^high^ monocytes infiltration ⬇	359
		Diet-induced NASH mice	High-fat diet containing 0.015% CVC for 12 weeks	M1-like macrophages ⬇, M2-like macrophages ⬆, hepatic stellate cell activation ⬇, hepatic lipid accumulation and peroxidation ⬇,	361
		Diet-induced NASH mice	30 mg/kg/d for 4 weeks or 30 mg/kg/d for 14 weeks, i.p.	Ly6C^high^ BM-derived macrophages accumulation ⬇, liver fibrosis ⬇	362
		Mdx mice (genetic homologue of DMD)	20 mg/kg/d, for 4 weeks, i.p.	Total infiltrating macrophages ⬇, disease progression ⬇	363
RS-102895	CCR2	Renal artery stenosis mice	10 mg/kg/d, for 4 weeks, i.g.	Renal atrophy ⬇, iNOS^+^ and CD206^+^ macrophage accumulation ⬇	371
RS-504393	CCR2	UUO mice	2 mg/kg twice a day, i.g.	Macrophage infiltration and activation ⬇	257
		BLM-induced scleroderma mice	4 mg/kg/d or 8 mg/kg/d, three days per week, for 4 weeks, i.d.	The number of mast cells and myofibroblasts in the skin ⬇, mRNA levels of TGF-β1 and collagen α1 ⬇,	373
		Rheumatic heart disease rats	5 mg/kg, once daily for 8 weeks, i.p.	Macrophage infiltration, inflammation, and fibrosis ⬇	207
		Bladder outlet obstruction rats	5 mg/kg, for 6 weeks, i.g.	Macrophage infiltration in bladder tissue ⬇, bladder fibrosis ⬇	374
NOX-E36	CCR2	Diabetic Apo E ^-/-^ mice	20 mg/kg, three times a week for 4 weeks, c	Albuminuria ⬇, CCR2-expressing Ly6C^high^ monocytes ⬇, restores glomerular endothelial glycocalyx and barrier function	376
		Mice with type 2 diabetes	50 mg/kg, three times per week, continued for 8 weeks, i.d.	Glomerular macrophages ⬇, diffuse glomerulosclerosis ⬇	377
		Mice with metabolic liver fibrosis	20 mg/kg NOX-E36, s.c.	Intrahepatic levels of proinflammatory cytokines ⬇, early influx of Ly6C^high^ MDMs ⬇, liver fibrosis ⬇	379
INCB334	CCR2	Ang II-treated mice	30 mg/kg/d, continued for 21 days, i.p.	Ly6C^high^ monocyte and M2 macrophage accumulation ⬇, aortic collagen deposition, elastin loss, and BP ⬇	382
SKL-2841	CCL2	BLM-induced scleroderma mice	100 mg/kg SKL-2841, for 3 days or 3 weeks, i.p.	Infiltration of inflammatory mononuclear cells and polymorphonuclear cells ⬇	286
OPL-CCL2-LPM	CCR2	Rats of anti-thymocyte serum-induced mesangioproliferative glomerulonephritis	50 or 100 microg/kg, from days 2, 4, 6, and 8, i.v.	CCR2^+^ MDMs ⬇, mesangial cell proliferation ⬇, ECM synthesis ⬇	383
Anti-CCL2 NAb	CCL2	Rats with suprarenal aortic constriction	2 mg/kg/d, daily from 1 day before operation to day 28, i.v.	Macrophage accumulation ⬇, fibroblast proliferation ⬇, TGF-β ⬇	202
Honokiol	CCL2	UUO rats	2.5 mg/kg, twice per day, for 14 days, i.g.	Tubulointerstitial fibrosis ⬇, expression of pro-fibrotic factors ⬇, accumulation of type I collagen and fibronectin ⬇	388
Tianhuang formula	CCL2/MAPK/NF-κB	CCl_4_-induced liver fibrosis mice	4.8 g/kg for 6 weeks, i.g.	Serum ALT, AST, and hepatic TG levels ⬇, expression of fibrosis and inflammation markers ⬇	389
Quercetin	ERK1/2-C/EBPβ	Mice with experimental autoimmune myocarditis	20mg/kg every other day, i.g.	CCL2 ⬇, Cardiac function ⬆, inflammation ⬇, fibrosis ⬇	390
Dachaihu decoction	CCL2	Mice with chronic pancreatitis	14 g/kg/d, intragastrically	Macrophages infiltration ⬇, degree of fibrosis ⬇, IL-6 and fibronectin levels ⬇	387
Fu-Gan-Wan	NF-κB/CCL2/CCR2	CCl_4_-induced liver fibrosis mice	9.75 g/kg or 19.5 g/kg, once a day for 4 weeks, i.g.	ALT and AST ⬇, collagen deposition ⬇, pro-fibrotic factors α-SMA, COL1α1, CTGF, TIMP-1, TGF-β1 ⬇	391
Puerarin	CCL2, CCL7	DSS-induced colitis mice	25 and 50 mg/kg/d	Restores intestinal barrier integrity, proinflammatory cytokine production ⬇, colitis ⬇	392
		MI mice	100 mg/kg/d for 28 days, i.p.	Monocytes/macrophages activation ⬇, TGF-β1 ⬇, cardiac fibrosis ⬇	394
Arctigenin	ROS/ERK1/2/ NF-κB	UUO rats	1 and 3mg/kg/d, for 11 consecutive days, by gastric gavage	CCL2 ⬇, macrophages infiltration ⬇, TNF-α ⬇, IL-1β ⬇, IFN-γ ⬇, TGF-β1 ⬇, oxidative stress and EMT of renal tubules ⬇	396
Astragalus	TGF-β1	Rats with peritoneal dialysis	1000/2000/4000 mg/kg/d for 7 d, i.p.	CCL2 ⬇, monocytes/macrophages activation⬇, TGF-β1 ⬇	397
AS-IV	NF-κB	LPS-induced H9C2 cells	AS-IV (5, 10, and 50 μM) groups	CCL2 ⬇, cell surface size ⬇, cardiac hypertrophy and fibrosis ⬇	399
		Streptozotocin-induced diabetic rats	5 and 10 mg/kg/d for 8 weeks, p.o.	Serum levels of TNF-α, CCL2 and ICAM-1 ⬇, type I collagen production ⬇	400
Curcumin	CCL2	Pancreatic stellate cells	Curcumin at 1, 2.5, 5,10, 25 mM	Inhibits IL-1β and TNF-α-induced activation of AP-1 and MAPK, CCL2 ⬇, α-SMA level ⬇, type I collagen production ⬇	401
		CCl_4_-induced liver fibrosis mice	200 mg/kg/d, once daily, for 4 weeks, i.g.	Ly6C^high^ monocytes intrahepatic infiltration ⬇, kupffer cells activation ⬇, pro-inflammatory and pro-fibrogenic cytokines ⬇	402
		CCl_4_-induced liver fibrosis mice	200 mg/kg body weight daily, for 6 weeks, i.g.	CCL2 ⬇, TNF-α and TGF-β1 ⬇, Gr1^hi^ monocytes infiltration ⬇, inflammation and fibrosis ⬇	403
		Mice with NASH	25 microg per mouse, every other day, i.p.	Intrahepatic gene expression of CCL2, CD11b, procollagen type I, α-SMA, and TIMP-1 ⬇, reactive oxygen species ⬇	404

Abbreviations: ALT, alanine aminotransferase; Ang II, angiotensin II; AP-1, activator protein 1; Apo E, apolipoprotein E; AST, aspartate aminotransferase; AS-IV, Astragaloside IV; BLM, bleomycin; BM, bone marrow; BP, blood pressure; CCl_4_, carbon tetrachloride; CCR2, C-C motif chemokine receptor 2; COL1α1, collagen type I α 1 chain; CTGF, connective tissue growth factor; CVC, cenicriviroc; DMD, duchenne muscular dystrophy; DSS, dextran sulfate sodium; ECM, extracellular matrix; EMT, epithelial-mesenchymal transition; ERK1/2, extracellular signal-regulated kinase 1/2; FSCN1, fascin actin-bundling protein 1; HERC6, HECT and RLD domain containing E3 ubiquitin protein ligase family member 6; ICAM-1, intercellular adhesion molecule 1; i.d., intradermal injection; IFN-γ, interferon-gamma; i.g., intragastric injection; IL-1β, interleukin-1 beta; iNOS, inducible nitric oxide synthase; i.p., intraperitoneal injection; i.v., intravenous injection; LPS, lipopolysaccharide; MAPK, mitogen-activated protein kinase; MDMs, monocyte-derived macrophages; MI, myocardial infarction; NASH, non-alcoholic steatohepatitis; NF-κB, p65 subunit of nuclear factor-κB; p.o., oral (by mouth); ROS, reactive oxygen species; s.c., subcutaneous injection; α-SMA, α-smooth muscle actin; TG, triglycerides; TGF-β, transforming growth factor β; TIMP-1, tissue inhibitor of metalloproteinases 1; TNF-α, tumor necrosis factor α; UUO, unilateral ureteral obstruction.

**Table 3:** Clinical trials of drugs targeting the CCL2/CCR2 axis in the treatment of fibrotic diseases.

Drug	Target	Experimental object	Research stage	Treatment outcomes and safety	Clinical limitations/reasons for failure	Trial identification	Refs
CVC	CCR2/CCR5	Patients with NASH and stage 0-4 liver fibrosis	II	CVC 150 mg once daily was well tolerated, with a median treatment duration of 33.6 months	/	NCT03059446	364
		Patients with at least 6 months of PSC	II	Treatment with CVC 150 mg for 24 weeks was well tolerated and a modest decrease (median of 18%) in the endpoint of ALP	The main limitations are the small sample size and single-arm study design, which means that each participant was his or her own control	NCT02653625	355
		NASH patients with NAFLD activity score at screening (4 or ≥5) and fibrosis stage (≤2 or >2)	IIb	Antifibrotic benefit with good tolerability at year 1, achieves improvement in fibrosis by ≥1 stage and no worsening of NASH	Differences in responses among subgroups (e.g., region, sex, and T2DM) that may reflect the multifactorial nature of the disease or be associated with sample size of the subgroups	NCT02217475	365
		Patients with NASH and stage 2 or 3 liver fibrosis	III	Safe and well tolerated, primary endpoint—fibrosis regression (22.3% in the CVC group vs. 25.5% in the placebo group) and no worsening of NASH was not met	CVC inhibits Ly6C^high^ macrophage infiltration but fails to eliminate pro-fibrotic TREM2⁺ scar-associated macrophages, thus failing to drive reversal of deposited collagen	NCT03028740	367
NOX-E36	CCL2	Type 2 diabetic patients with albuminuria	IIa	Generally safe and well tolerated, the urinary albumin/creatinine ratio (ACR) from baseline to week 12 decreased by 29% (P < 0.05)	Baseline characteristics are not balanced between the groups due to small sample size, which contributes to a worse renal and metabolic outcome in the placebo group	NCT01547897	422
Carlumab	CCL2	Patients with progressive IPF disease within 12 months	II	The primary endpoint of percentage change in forced vital capacity did not demonstrate any therapeutic effect. SGRQ scores indicated a slight trend toward worsening during treatment.	The primary cause of failure is the excessive activation of compensatory mechanisms under CCL2 inhibition, which also involves pharmacodynamic limitations and patient population heterogeneity.	NCT00786201	385

Abbreviation: ALP, alkaline phosphatase; ACR, albumin/creatinine ratio; CVC, cenicriviroc; CCR2, C-C motif chemokine receptor 2; IPF, idiopathic pulmonary fibrosis; NAFLD, non-alcoholic fatty liver disease; NASH, non-alcoholic steatohepatitis; PSC, primary sclerosing cholangitis; T2DM, type 2 diabetes mellitus; TREM2, triggering receptor expressed on myeloid cells 2.

## Abbreviations

AECs: alveolar epithelial cells
ADGRF5: adhesion G-protein coupled receptor F5
AKT: protein kinase B
AP-1: activating protein-1
Ang-II: angiotensin II
AS-IV: astragalus saponin IV
α-SMA: α-smooth muscle actin
ASH1L: ASH1-like histone lysine methyltransferase
BALF: bronchoalveolar lavage fluid
CCL2/MCP-1: monocyte chemotactic protein-1
CCR2: C-C motif chemokine receptor 2
CCR2 ^-/-^: CCR2 knockout
CFs: cardiac fibroblasts
CHF: congestive heart failure
CKD: chronic kidney disease
CREB: cyclic AMP response element-binding protein
cRM: cardiac resident macrophages
CVC: cenicriviroc
Cytl1: cytokine-like 1
DMD: Duchenne muscular dystrophy
DN: diabetic nephropathy
DSS: dextran sulfate sodium
EAO: Experimental autoimmune orchitis
ECs: endothelial cells
ECM: extracellular matrix
EMT: epithelial-mesenchymal transition
ERK: extracellular-signal-regulated kinase
ESRD: end-stage renal disease
Ezh2: enhancer of Zeste homolog 2
ET: essential thrombocythemia
FITC: fluorescein isothiocyanate
FOXF1: forkhead box F1
GM-CSF: granulocyte-macrophage colony-stimulating factor
GPCRs: G protein-coupled receptors
HCV: hepatitis C virus
HIF-1α: hypoxia-inducible factor-1α
H3K4me3: Histone H3 lysine 27 trimethylation
HSCs: hepatic stellate cells
IFN-γ: interferon-γ
IL-1β: interleukin-1β
ILD: interstitial lung disease
IPF: idiopathic pulmonary fibrosis
I/R: ischemia-reperfusion injury
JAK: janus kinase
JNK: c-Jun N-terminal kinase
LPS: lipopolysaccharide
LRRK2: leucine-rich repeat kinase 2
MAPK: mitogen-activated protein kinase
MDMs: monocyte-derived macrophages
MEK: mitogen-activated protein kinase kinase
MI: myocardial infarction
MMPs: matrix metalloproteinases
MPN: myeloproliferative neoplasms
MSMP: microseminoprotein
mTOR: mammalian target of rapamycin
NADPH: nicotinamide adenine dinucleotide phosphate
NAFLD: non-alcoholic fatty liver disease
NASH: non-alcoholic steatohepatitis
NETs: neutrophil extracellular traps
NFAT: activator nuclear factor of activated T-cells
NF-κB: p65 subunit of nuclear factor-κB
NK: natural killer
PGE2: prostaglandin E2
PH: pulmonary hypertension
PIP2: phosphatidylinositol bisphosphate 2
PI3K: phosphoinositide 3-kinase
PKC: protein kinase C
PSCs: pancreatic stellate cells
PSMP: PC3-secreted microprotein
PV: polycythemia vera
SCF: stem cell factor
scRNA-seq: single-cell RNA sequencing
SNPs: single nucleotide polymorphisms
Sp1: specific protein 1
SSc: systemic sclerosis
STAT: signal transducer and activator of transcription
TIMP-1: tissue inhibitor of metalloproteinase 1
TMAO: trimethylamine N-oxide
TNF-α: tumor necrosis factor α
Tregs: regulatory T cells
Tsk-1: tight skin 1
TSLP: thymic stromal lymphopoietin
3'UTRs: 3' untranslated regions
UUO: unilateral ureteral obstruction
WT: wild-type
